# Causal association between sarcopenia-related traits and osteoarthritis: A bidirectional 2-sample Mendelian randomization Study

**DOI:** 10.1097/MD.0000000000043069

**Published:** 2025-07-11

**Authors:** Cheng Zhang, Binglang Xiong, Yuhang Shi, Tianzhao Tian, Rongtian Wang, Guangyi Zhang, Haijun He, Weiheng Chen

**Affiliations:** a Department of Orthopedic, Wangjing Hospital of China Academy of Chinese Medical Sciences, Beijing, China; b Department of Orthopedic, The Third Affiliated Hospital of Beijing University of Chinese Medicine, Beijing, China; c Department of Orthopedic, Hospital of Traditional Chinese Medicine Affiliated to Guangzhou Medical University, Guangzhou, China; d Department of Orthopedic, Wangjing Clinical Medical College, Beijing University of Chinese Medicine, Beijing, China.

**Keywords:** causal relationship, hip osteoarthritis, knee osteoarthritis, Mendelian randomization, sarcopenia

## Abstract

Accumulating evidence from observational studies indicated that sarcopenia and osteoarthritis (OA) may interact in pathomechanism. Therefore, the present 2-sample Mendelian randomization (MR) study aimed to reveal the bidirectional causal association between sarcopenia-related traits and OA. We extracted instrumental variables strongly associated with sarcopenia-related traits, namely low grip strength, appendicular lean mass, and usual walking pace from 3 large-scale genome-wide association studies involving 256,523, 450,243, and 459,915 individuals, respectively. Summary-level data for knee and hip OA were obtained from a genome-wide association studies meta-analysis conducted by the UK Biobank and arcOGEN, involving 455,221 individuals of European descent. The inverse-variance weighted (IVW) method was utilized as primary MR analysis, whereas the weighted median, MR-Egger regression, and MR pleiotropy residual sum and outlier (MR-PRESSO) were performed as complementary methods to verify the robustness of findings. Our findings indicated genetically predicted usual walking pace was inversely associated with hip OA (IVW OR = 0.30, 95 CI = 0.17–0.53, *P* = 4.27 × 10^−5^) as well as knee OA (IVW OR = 0.20, 95 CI = 0.12–0.33, *P* = 1.07 × 10^−9^). In the reverse MR analyses, genetically predicted hip OA (IVW beta = −0.027, 95 CI = −0.038, −0.016, *P* = 6.83 × 10^−7^) demonstrated a negative causal effect on usual walking pace, while knee OA (IVW beta =0.002, 95 CI = −0.031 to 0.035, *P* = .898) did not show a significant effect. However, no evidence was found to suggest a causal effect of low grip strength and appendicular lean mass on hip or knee OA, and vice versa. Our study suggests a negative causal effect of usual walking pace, a sarcopenia-related traits, on knee and hip OA, with hip OA also negatively affecting walking pace. Further research is needed to explore the mechanisms linking sarcopenia-related traits and site-specific OA, aiming to identify common therapeutic targets.

## 1. Introduction

Osteoarthritis (OA) is a disease involving the whole joint. The degradation of the cartilage is intimately associated with subchondral bone remodeling, synovial membrane inflammation, and injury to the surrounding muscles.^[1]^ It is reported there are currently more than 300 million individuals worldwide affected by OA, with a prevalence of more than 13.4% among adults in the United States.^[2]^ Furthermore, this prevalence is increasing annually due to the aging population and the global rise in obesity rates. More importantly, as the disease advances to its late stages, it impairs mobility and indirectly raise the risk of cardiovascular disease and mortality.^[3,4]^ As we all know, there is a lack of effective prevention and treatment options for early and middle stages of OA (especially hip and knee OA), apart from joint replacement surgery in advanced cases. Therefore, it is crucial to explore potential risk factors and eliminate predisposing variables for the early prevention and treatment of OA.

The concept of sarcopenia was initially proposed in 1989 and has since gained significant attention over the past decade. It is characterized by a decline in muscle mass and strength, often accompanied by reduced physical function.^[5]^ Similar to OA, sarcopenia is also a degenerative musculoskeletal disease that develops with aging, and the 2 conditions frequently coexist in middle-aged and elderly women, which is the leading cause of falls and fractures in the elderly.^[6,7]^ Systemic inflammation and fluctuating hormone levels (estrogen and testosterone) may be the common pathological mechanisms of the 2 diseases.^[8]^ Strong muscles contribute to joint stability and serve a protective function. However, when muscle strength declines due to muscle atrophy, this protective effect diminishes as well. Likewise, patients with OA may also experience disuse atrophy of muscles around the affected joints due to prolonged pain and limited mobility. Results from a large population-based longitudinal cohort study suggested that knee OA was associated with decreased quadriceps cross-sectional area and increased intramuscular adipose tissue, which in turn predicted later symptom worsening and an increased risk of knee arthroplasty.^[9]^ Several large-scale cross-sectional studies have also found that the presence of sarcopenia increases the severity of OA and the intensity of the pain.^[10,11]^ Conversely, a multicenter longitudinal cohort study in the United States suggested that sarcopenic obesity, rather than sarcopenia alone, contributes to the risk of knee OA.^[12]^ However, the true causal relationship between sarcopenia and OA has not been systematically investigated due to potential biases of traditional observational studies, including confounding factors and reverse causation.

Mendelian randomization (MR) is a novel method that utilizes genetic variants as instrumental variables(IVs)to infer causal associations between exposures and outcomes.^[13]^ The underlying idea of MR is that genetic variants are randomly assigned during conception and typically not associated with other traits.^[14]^ This procedure is similar to the randomization of participants to treatment and control groups in a randomized controlled trial. Based on this, MR studies minimize the likelihood of reverse causality since alleles are fixed at birth and cannot vary with the onset or progression of a disease. Furthermore, based on the availability of detailed phenotype of conditions from large-scale genome-wide association studies (GWAS), MR could further explore the causal relationship between more disease phenotypes.

In present 2-sample bidirectional MR Study, the bidirectional causal effect of 3 sarcopenia-related phenotypes-low grip strength, appendicular lean mass (ALM), and usual walking pace on OA was analyzed.

## 2. Methods

### 2.1. Study design

**Figure 1.**
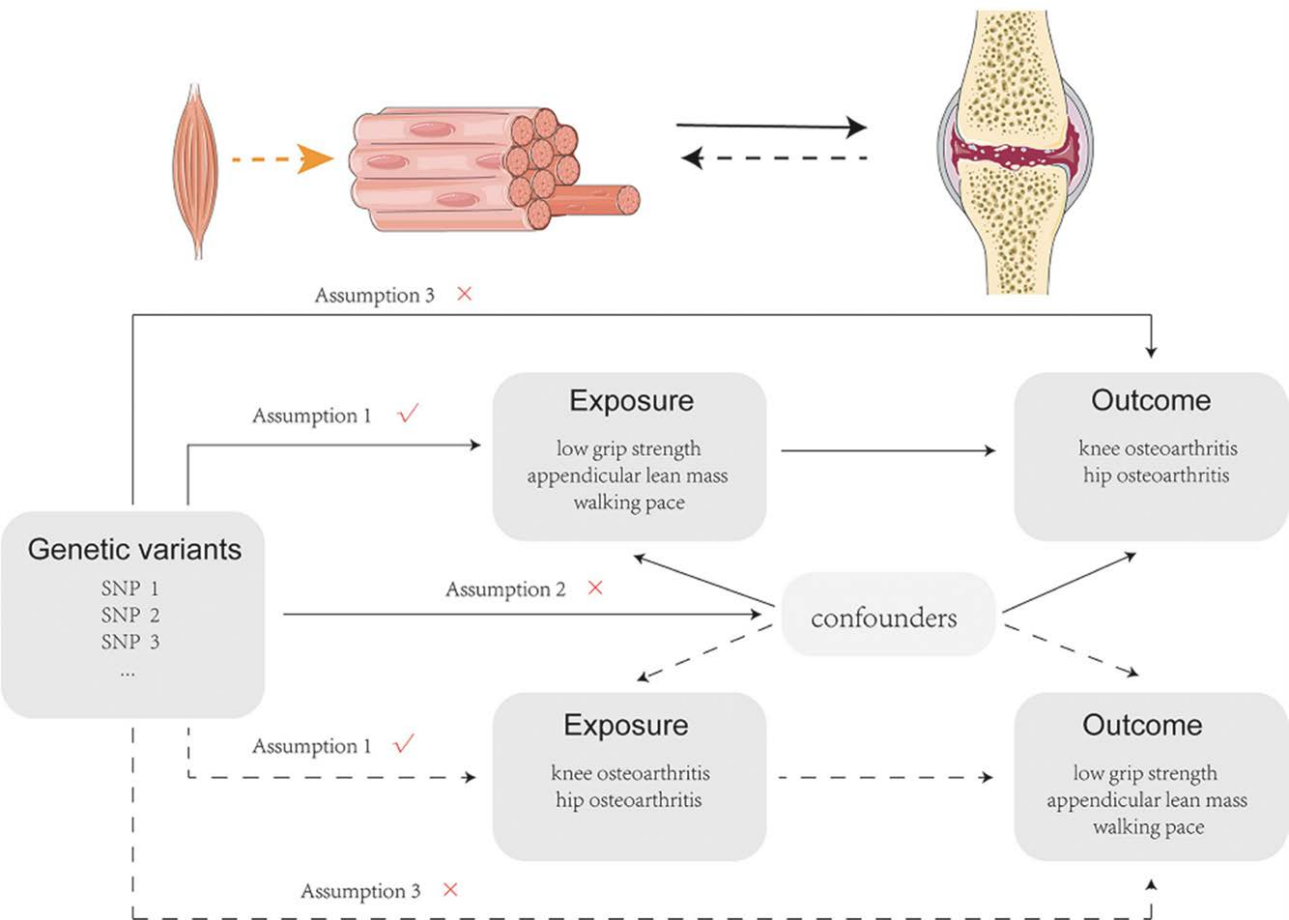
Schematic diagram of the present bidirectional Mendelian randomization (MR) design. The solid arrows depict the pathway of sarcopenia-related traits exerting effect on hip OA and knee OA using genetic variants strongly associated with exposure, and the dotted arrows show the reverse analysis. OA = osteoarthritis, SNPs = single-nucleotide polymorphisms.

Figure 1 shows a schematic diagram of the present bidirectional MR design. This bidirectional MR analysis was conducted in 2 steps: sarcopenia-related traits were selected as exposures while knee OA and hip OA were selected as outcomes in the first step, whereas the second step was reversed. The 3 following assumptions must be achieved by MR analysis: Firstly, genetic variants used as IVs are strongly associated with exposure. Secondly, the IVs are not associated with potential confounders. Thirdly, IVs can affect the outcome only through the exposure rather than other pathways.

### 2.2. Data sources and SNP selection for sarcopenia

Sarcopenia is usually assessed by muscle strength, muscle mass, muscle quality and physical function. So in this study, we selected low grip strength, ALM and usual walking pace as the related phenotypes of sarcopenia. We obtained the summary-level data of low grip strength from a meta‐analysis of muscle weakness GWAS performed by the CHARGE consortium involving a total of 256,523 individuals of European descent aged 60 years or older (48,596 cases vs 207,927 controls).^[15]^ Low grip strength in this GWAS was defined as <30 kg for men and 20 kg for women using the European Sarcopenia Working Group criteria.^[5]^ We selected the summary-level data for ALM from a large GWAS with 450,243 UK Biobank cohort participants, identifying 1059 independent variants from 799 loci at the genome-wide significance level.^[16]^ Besides, genetic associations with usual walking pace were assessed by the GWAS summary-level statistics from the UK Biobank, which includes 459,915 individuals of European ancestry (https://gwas.mrcieu.ac.uk/datasets/ukb-b-4711/). Based on the 3 assumptions of MR study, single-nucleotide polymorphisms (SNPs) associated with low grip strength, ALM and usual walking pace at genome-wide significance (*P* < 5 × 10^−8^) were extracted. Meanwhile, an independent test was performed to prevent the linkage disequilibrium among these SNPs (r^2^ < 0.001). Following that, we searched in the Phenoscannner database (https://www.ensembl.org) to determine whether these SNPs(*P* < 1 × 10^−5^) were associated with any potential confounders (OA-related traits), such as BMI, body fat percentage, type-2 diabetes and obesity.^[17]^ SNPs associated with confounders above were manually removed to avoid possible pleiotropic effects (Table S1, Supplemental Digital Content, https://links.lww.com/MD/P381). Furthermore, the *F*-statistics and *R*^2^ of each SNP was calculated to qualify the strength of IVs, SNPs with a *F*-statistics <10 were considered weak instruments and would be omitted from MR analysis. Finally, 12SNPs, 516SNPs, 40SNPs were taken as IVs for low grip strength, ALM, and usual walking pace, respectively (Table S2, Supplemental Digital Content, https://links.lww.com/MD/P382).

### 2.3. Data sources and SNP selection for osteoarthritis

Summary statistics data of knee OA and hip OA were available from the largest GWAS meta-analysis conducted by the UK Biobank and the Arthritis Research UK Osteoarthritis Genetics (arcOGEN) with 455,221 European population participants, including 24,955 cases with knee OA, 15,704 cases with hip OA and 378,169 controls.^[18]^ Details of all GWASs included in our study are represented in Table 1. Similarly, we extracted SNPs associated with knee OA and hip OA at genome-wide significance (*P* < 5 × 10^−8^) and remained independent SNPs by linkage disequilibrium test (*r*^2^ < 0.001). Furthermore, we eliminated SNPS with *F*-statistic lower than 10 or those associated with confounders (Sarcopenia-related traits), such as type-2 diabetes, hyperthyroidism, hypothyroidism (Table S1, Supplemental Digital Content, https://links.lww.com/MD/P381). Ultimately, 8SNPs, 24SNPs were taken as IVs for knee OA and hip OA, respectively (Table S3, Supplemental Digital Content, https://links.lww.com/MD/P383).

**Table 1:** Details of the GWASs included in present study.

Phenotype	Consortium	No. SNPs	Sample size	Population
Low grip strength	CHARGE	9,336,415	256,523 (48,596 cases, 207,927 controls)	European
ALM	UK Biobank	18,071,518	450,243	European
Usual walking pace	UK Biobank	9,851,867	459,915	European
Knee OA	UK Biobank and arcOGEN	29,999,696	403,124 (24,955 cases, 378,169 controls)	European
Hip OA	UK Biobank and arcOGEN	29,771,219	393,873 (15,704 cases, 378,169 controls)	European

ALM = appendicular lean mass, OA = osteoarthritis, SNPs = single-nucleotide polymorphisms.

### 2.4. Statistical analysis

After harmonizing the effect alleles across the IVs of exposure and outcome, we employed the inverse-variance-weighted (IVW) as the primary MR analysis method which pools the Wald ratio estimates for the causal effect of each SNP.^[19]^ The IVW provides the most precise estimates under the premise that all SNPs are valid. Several sensitivity analyses were performed, including the weighted median (WM), MR-Egger regression and MR pleiotropy residual sum and outlier (MR-PRESSO) method, to ensure the robustness of IVW and detect possible pleiotropy. Among them, the WM method selects the median value to estimate the causal effect, which can provide consistent results on the basis that at least 50% of the genetic variations are valid IVs.^[20]^ MR-PRESSO test can detect outliers and perform causality analysis after removing outliers.^[21]^ The MR-Egger method relaxed the requirement that there be no pleiotropy between genetic variants in the IVW method, it can detect and adjust for the directional pleiotropic effect but exhibited low precision.^[22]^ Directional pleiotropy was evaluated by the MR-Egger intercept test (*P* for intercept < .05). Furthermore, we implemented the Cochran *Q* statistic to assess the statistical heterogeneity among SNPS, random-effects model of IVW was used if *P* < .05. Leave-one-out test was also performed to assess whether the causal effect could be biased by a single SNP by ruling out each SNP one by one.

In view of the multiple testing performed in this study, Bonferroni-corrected threshold at *P* < .004 (0.05/3 [sarcopenia-related traits]*2 [knee OA, hip OA]*2 [bidirectional]) was considered statistically significant. The results of MR analysis were presented as odds ratios (OR) or beta with corresponding 95% confidence intervals, when outcomes were binary (low grip strength, knee OA, hip OA) or continuous (ALM and usual walking pace) respectively. MR analyses were performed using the “TwoSample MR” package and forest plots were generated using “forestploter” package in R project (4.22 version).

### 2.5. Ethics

Since this MR study is based on publicly available GWAS summary statistics, additional ethical approval was not needed.

## 3. Results

### 3.1. Causal effect of sarcopenia-related traits on osteoarthritis

In total, 12 SNPs, 516 SNPs, 40 SNPs were taken as IVs for low grip strength, ALM and usual walking pace, respectively. Detailed description of the IVs above are available in Table S2, Supplemental Digital Content, https://links.lww.com/MD/P382. As shown in Table S2, Supplemental Digital Content, https://links.lww.com/MD/P382, the *F*-statistics for all IVs exceeded 10, indicating the absence of bias caused by weak IVs. To fulfill the third assumption of MR that the IVs cannot be directly associated with the outcome, SNPS strongly associated with each outcome (hip OA, knee OA) at the genome-wide significance threshold (*P* < 5 × 10^−8^) were removed. Additionally, SNPs with incompatible and palindromic alleles were further omitted from the harmonizing process prior to MR Analysis.

**Figure 2.**
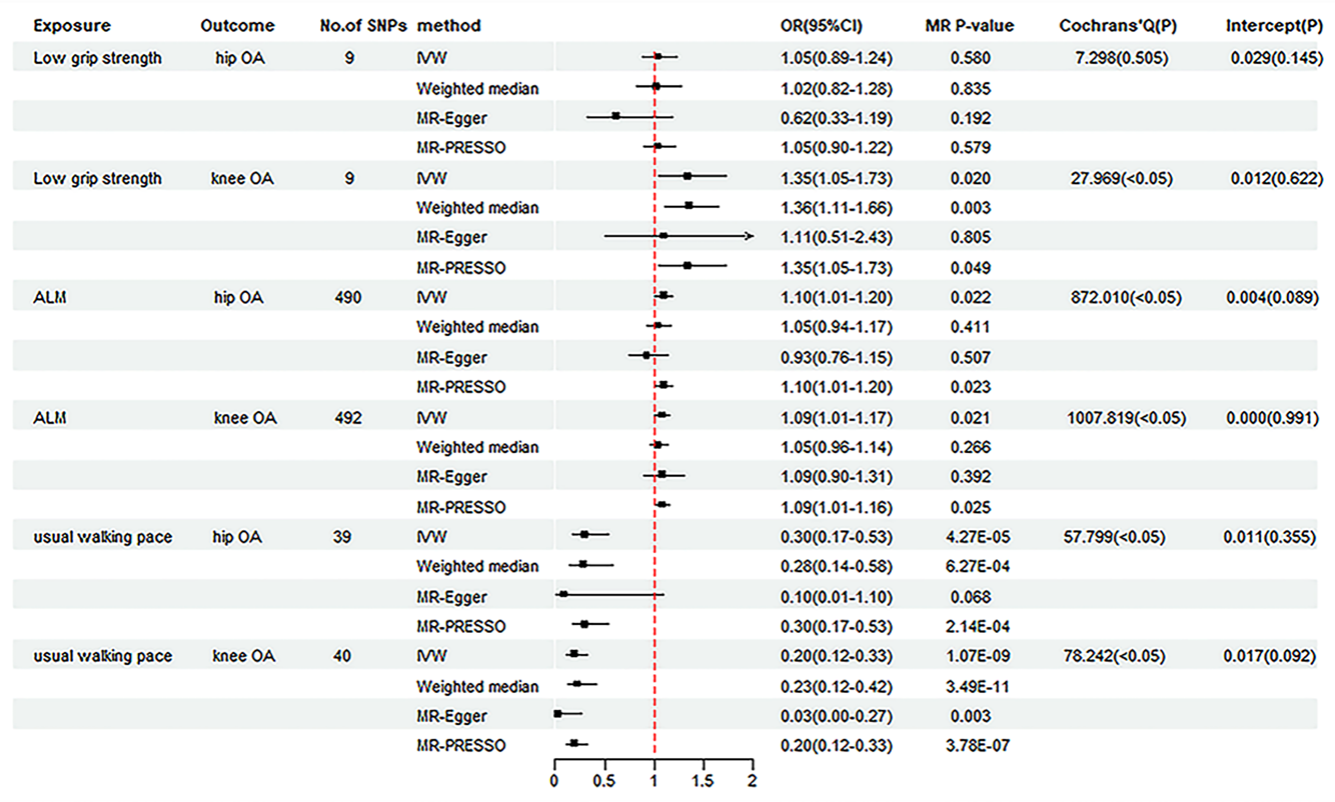
Forest plot of MR results for the causal effect of sarcopenia-related traits on hip OA and knee OA. ALM = appendicular lean mass, CI = confidence interval, IVW = inverse-variance-weighted, MR = Mendelian randomization, OA = osteoarthritis, OR = odds ratios, PRESSO = pleiotropy residual sum and outlier, SNPs = single-nucleotide polymorphisms.

As shown in Figure 2, the IVW results suggested that genetically predicted usual walking pace had a negative causal effect on hip OA (IVW OR = 0.30, 95 CI = 0.17–0.53, *P = *4.27 × 10^−5^) and knee OA (IVW OR = 0.20, 95 CI = 0.12–0.33, *P = *1.07 × 10^−9^). The causal effects were also robust across other complementary MR analyses like WM and MR-PRESSO. No causal effect was detected between genetic liability for low grip strength and hip OA (IVW OR = 1.05, 95 CI = 0.89–1.24, *P = *.580), knee OA (IVW OR = 1.35, 95 CI = 1.05–1.73 *P = *.020). The evidence provided by MR-Egger and MR-PRESSO supported the findings of the IVW analysis, while the WM analysis for low grip strength-knee OA presented a significant result (OR = 1.36, 95 CI = 1.11–1.66 *P = *.003). Similarly, genetically predicted that ALM had no causal effect on hip OA (IVW OR = 1.10, 95 CI = 1.01–1.20, *P = *.022) and knee OA (IVW OR = 1.09, 95 CI = 1.01–1.17, *P = *.021), and other MR analysis methods yielded consistent results with IVW. The scatter plots displaying the causal effect of sarcopenia-related traits on hip and knee OA are presented in Figure S1, Supplemental Digital Content, https://links.lww.com/MD/P380.

Although heterogeneity was detected in many of the results above by Cochran *Q* test, a random-effects IVW was adopted to balance of heterogeneity to ensure reliability of the results (Fig. 2). ALL the *P*-values from the MR-Egger intercept test were >.05, suggesting the absence of pleiotropy (Fig. 2). Moreover, leave-one-out analysis indicated that the causal effect of usual walking pace on hip OA and knee OA was not driven by any single SNP (Fig. S2, Supplemental Digital Content, https://links.lww.com/MD/P380).

### 3.2. Causal effect of osteoarthritis on sarcopenia-related traits

Similarly, 24 SNPs and 8 SNPs were taken as IVs for hip OA and knee OA separately (Table S3, Supplemental Digital Content, https://links.lww.com/MD/P383). The *F*-statistics for IVs were all above the threshold of 10 (Table S3, Supplemental Digital Content, https://links.lww.com/MD/P383). SNPS strongly associated with each outcome (low grip strength, ALM and usual walking pace) were further removed (*P* < 5 × 10^−8^).

**Figure 3.**
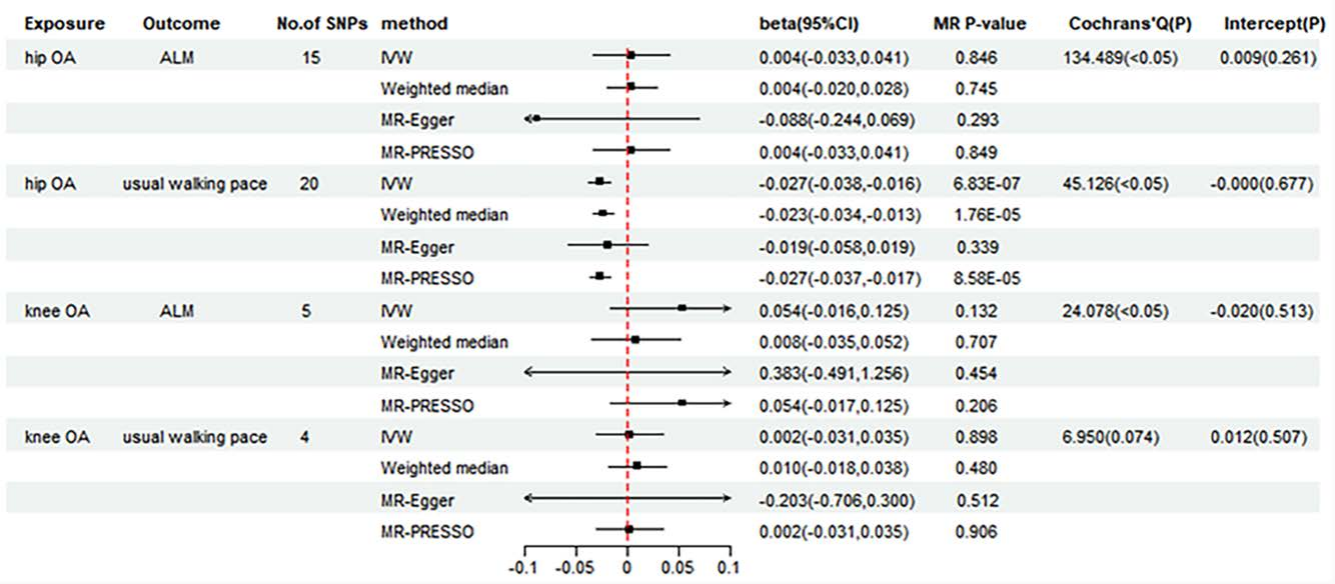
Forest plot of MR results for the causal effect of hip OA and knee OA on ALM and usual walking pace. ALM = appendicular lean mass, CI = confidence interval, IVW = inverse-variance-weighted, MR = Mendelian randomization, OA = osteoarthritis, PRESSO = pleiotropy residual sum and outlier, SNPs = single-nucleotide polymorphisms.

**Figure 4.**
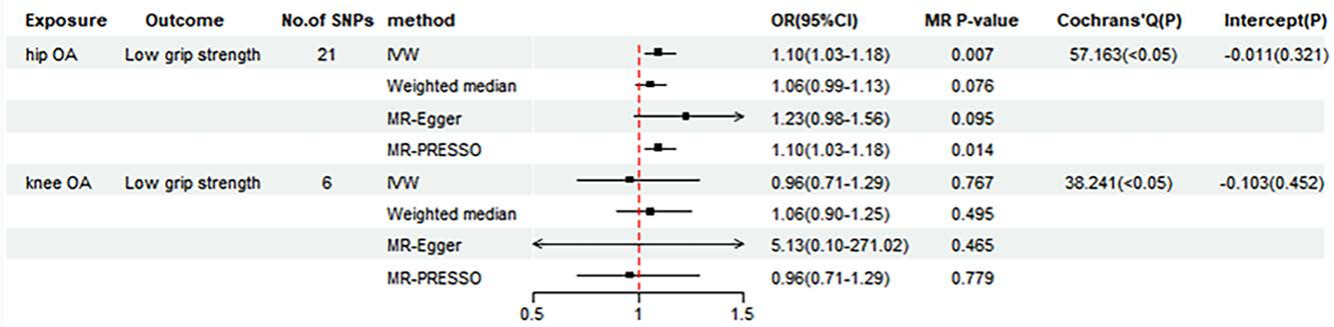
Forest plot of MR results for the causal effect of hip OA and knee OA on low grip strength. ALM = appendicular lean mass, CI = confidence interval, IVW = inverse-variance-weighted, MR = Mendelian randomization, OA = osteoarthritis, OR = odds ratios, PRESSO = pleiotropy residual sum and outlier, SNPs = single-nucleotide polymorphisms.

As shown in Figure 3, genetically determined hip OA was negatively associated with usual walking pace (IVW beta = −0.027, 95 CI = −0.038 to −0.016, *P = *6.83 × 10^−7^). This causal effect was similarly confirmed by the analyses of WM and MR-PRESSO. However, no genetic association was detected between hip OA and either low grip strength or ALM (Figs. 3 and 4). In addition, we did not observe any causal association between knee OA and low grip strength, ALM, usual walking pace in genetic predisposition (Figs. 3 and 4). The scatter plots illustrating the causal effect of hip and knee OA on sarcopenia-related traits are presented in Figure S3, Supplemental Digital Content, https://links.lww.com/MD/P380.

Similarly, we adopted the IVW method in a random-effects model to mitigate potential bias due to heterogeneity (Figs. 3 and 4). For all the estimates, MR-Egger intercept test did not detect any pleiotropy (Figs. 3 and 4). No single SNP substantially violated the causal effect of hip OA on usual walking pace in the leave-one-out sensitivity analysis (Fig. S4, Supplemental Digital Content, https://links.lww.com/MD/P380).

## 4. Discussion

In the current study, we investigated potential causal associations between sarcopenia-related phenotypes and OA using large-scale GWAS data. The results suggest that genetically predicted usual walking pace is negative associated with the risk of knee OA and hip OA. In the opposite direction, hip OA also exerts an adverse causal effect on usual walking pace.

Previous observational studies have identified sarcopenia as a potential independent risk factor for OA.^[23,24]^ A retrospective cohort study involving 2492 participants found that an initial diagnosis of sarcopenia was associated with a higher risk of developing symptomatic OA (OR = 2.29, 95% CI = 1.42–3.71, *P* = .001) after 4 years of follow-up. However, no significant association was observed between sarcopenia and the risk of incident radiographic OA (OR = 1.48, 95% CI = 0.53–4.10, *P* = .45).^[25]^ In our present MR Study, we found that only usual walking pace, one of the sarcopenia-related traits, had a causal effect on knee OA and hip OA. Considering that low grip strength and ALM are also the reference criteria for diagnosing sarcopenia, our results implies that the causality between sarcopenia and OA was only partially supported. One probable explanation is that both knee and hip are weight-bearing joints in the lower extremities, which are more susceptible to the periarticular muscle mass and strength (e.g., quadriceps and hamstrings), rather than hand grip strength and ALM. Regrettably, due to the lack of relevant GWAS data, we failed to focus on the causal relationship of quadriceps strength and muscle mass with knee OA and hip OA in present MR Study. Data from Korean National Health and Nutrition Survey, which included 4924 community residents, revealed that a low skeletal muscle mass index specifically in the lower limbs, rather than the entire body, independently affected knee OA.^[26]^ Moreover, a recent meta-analysis, including 46,819 participants with knee OA excluded at baseline, found a strong association between knee extensor weakness and the subsequent development of symptomatic knee OA as well as radiographic knee OA.^[27]^ Overall, these findings suggest that the causality between sarcopenia and OA may be more prominent for a specific joint and its surrounding muscles.^[28,29]^ In fact, muscles can only function when innervated by nerves. As a result, the usual walking pace can serve as a reliable indicator of lower limb function as it comprehensively reflect lower limb muscular strength and nervous system performance. Saulo et al also reported that the maximal quadriceps isometric strength is an important factor influencing the gait ability of knee OA patients.^[30]^ Another reason may bias the real association between sarcopenia and OA is the confounding factors, since sarcopenia tends to coexist with obesity in postmenopausal women, known as sarcopenic obesity (low muscle mass with high fat mass).^[31–33]^ Several large cohort studies have demonstrated that sarcopenic obesity has a more significant impact on OA than either sarcopenia or obesity alone.^[12,24]^ However, we have removed SNPs associated with obesity, body fat percentage, and diabetes during the screening process for IVs to minimize the effect of confounding variables.

As for the causal effect of OA on sarcopenia, accumulating evidence from traditional studies indicated that OA may contribute to the formation and progression of sarcopenia.^[34,35]^ A survey of German community residents aged 70 years or older found that participants with hip or lower limb OA had a 5.6% higher incidence of sarcopenia than those without OA.^[36]^ Chronic pain derived from OA could inhibit the neuronal activity at the neuromuscular junction, resulting in less muscle activity and disuse atrophy, eventually leading to sarcopenia.^[37,38]^ Moreover, multiple gait analysis studies have indicated that individuals with OA of the lower extremities tend to have an unsteady gait and a significantly slower walking pace due to decreased proprioceptive sensation and muscular strength.^[39,40]^ Similarly, in our present MR analysis, we found a negative causal effect of hip OA on usual walking pace, but no evidence of a causal relationship between knee OA and usual walking pace, nor any association between either knee OA or hip OA and low grip strength or ALM.

The underlying mechanism of the interaction between sarcopenia and OA may be intricate, since both are degenerative musculoskeletal diseases and share common risk factors, such as age, estrogen levels, inflammation, and obesity.^[8,28]^ From a biomechanical perspective, muscle atrophy can lead to joint instability, accelerating cartilage wear and OA degeneration, while the pain and limited mobility caused by OA will in turn worsen muscle atrophy, forming a vicious circle.^[41,42]^ Thus, it is important to recognize the tight relationship between the 2 diseases and focus on the prevention of the other when 1 disease develops. In view of this mechanism, researchers recommend a combination of resistance training and aerobic exercise to enhance knee extensor muscle strength, decrease body fat percentage, alleviate the pain of mild OA, and yield favorable outcomes.^[43–46]^ However, the long-term effect of muscular training in slowing cartilage degeneration is still being debated, despite its potential for symptom relief. At the cellular and molecular levels, myoblasts and chondrocytes may share common pathological targets and pathways. NF-kappa B (NF-κB) has been reported to be a crucial signaling pathway implicated in the pathophysiology of sarcopenia and OA.^[8,29,47]^ With aging, alterations in sex hormone levels, and increase in adipose tissue, circulating inflammatory cytokines such as IL-1, IL-6, and TNF-α reach a higher level, which upregulate the NF-κB pathway and contribute to increased proteolysis of muscle protein.^[48]^ On the other hand, these pro-inflammatory cytokines can directly act on the extracellular matrix of cartilage by activating the NF-κB pathway to promote the degradation of cartilage matrix.^[49,50]^ That is, systemic inflammation seems to a shared pathophysiological origin between OA and sarcopenia. Furthermore, aside from systemic inflammation, myocytes and chondrocytes could also produce cytokines and engage in paracrine communication due to their adjacent anatomical location.^[7,28,51]^ Based on the molecular mechanisms above, although several novel interventions, such as inflammatory factor antagonists, hormone replacements and Mediterranean diet, et., have emerged in recent years, their exact efficacy needs to be validated through numerous high-quality studies.^[52–54]^ Summarily, only a deeper understanding of the common pathological mechanisms underlying sarcopenia and OA would enable us to identify effective common therapeutic targets for both diseases.

Our study possesses several strengths. The primary strength lies in the implementation of MR design, which effectively mitigated the residual confounding and reverse causality inherent in observational studies, and the combination of multiple MR methods as well as sensitivity analysis have also furnished reliable evidence for the genetic association between sarcopenia and OA. Another strength is that IVs for both OA and sarcopenia-related traits were derived from the large sample summary-level GWAS, providing more accurate estimation results. Notwithstanding, some limitations should also be noted. Firstly, due to the lack of relevant GWAS, we failed to reveal the true genetic association of lower limb muscle strength and muscle mass with knee or hip OA independently. Future corresponding GWAS are expected to be published to enhance our comprehension of the interplay between muscles and adjacent joints. Secondly, since 1 single SNP can be associated with multiple phenotypes, although we have removed SNPS associated with several confounders that may affect sarcopenia and OA, we remain uncertain about the presence of other potential confounders that might still work through the second assumptions of MR. Thirdly, it is important to note that the UK Biobank data, from which the IVs for ALM and usual walking pace, as well as partial data on diagnosed osteoarthritis cases were sourced, may harbor potential biases. The participants in the UK Biobank are predominantly of European descent, limiting the generalization of our findings to other populations, such as Asians and Americans. Despite these shortcomings, the present study is the first to provide genetic evidence of causality between sarcopenia-related phenotypes and OA using a bidirectional 2-sample MR approach.

## 5. Conclusions

Using large-scale summary genetic data, our study strengthened the evidence that usual walking pace, one of the sarcopenia-related phenotypes, has a negative causal effect on knee OA as well as hip OA, and hip OA in turn exerts a negative effect on usual walking pace. More researches are warranted to investigate the underlying mechanisms of the intrinsic link between various sarcopenia traits and site-specific OA, aiming to identify shared therapeutic targets for both conditions.

## Acknowledgments

We acknowledge the CHARGE consortium, UK Biobank and arcOGEN for providing summary statistic data used in this study. We thank all participants in the GWAS for their contributions to the present study.

## Author contributions

**Conceptualization:** Cheng Zhang.

**Data curation:** Binglang Xiong.

**Formal analysis:** Binglang Xiong.

**Funding acquisition:** Weiheng Chen.

**Investigation:** Cheng Zhang.

**Methodology:** Cheng Zhang.

**Project administration:** Yuhang Shi, Tianzhao Tian.

**Software:** Rongtian Wang.

**Supervision:** Guangyi Zhang.

**Validation:** Binglang Xiong.

**Visualization:** Cheng Zhang.

**Writing – review & editing:** Haijun He.

## Supplementary Material


