# Supplementary material for: Causal association between sarcopenia-related traits and osteoarthritis: A bidirectional 2-sample Mendelian randomization Study
Source: Medicine (Baltimore). 2025 Jul 11;104(28):e43069. doi: 10.1097/MD.0000000000043069 (PMC12263018; doi:10.1097/MD.0000000000043069)
Supplement: Supplementary file 1 [file medi-104-e43069-s001.docx]

**Supplementary Table 1. The removed IVs due to confounders**

| Exposures | Confounders | The removed IVs |  |
| --- | --- | --- | --- |
| low‐grip strength | body fat percentage/type-2 diabetes | rs10952289, rs13107325, rs3118903, rs34415150 | |
| ALM | BMI/ body fat percentage/ type-2 diabetes /obesity | rs10019221, rs10119967, rs10202701, rs1047891, rs10491967, rs1051952, rs10796828, rs10807137, rs10849576  rs1093086, rs10995566, rs11042717, rs111622870, rs11187838, rs111901094, rs115912456, rs12325539, rs12463908  rs12533452, rs1260326, rs12714414, rs12761076, rs12882130, rs12894822, rs12997625, rs13041213, rs13112742  rs1317349, rs1472852, rs1487441, rs1608113, rs1662842, rs17036160, rs17496249, rs1786263, rs1903002  rs2052478, rs2070598, rs2112617, rs2165772, rs2194411, rs2287821, rs2436772, rs2524139, rs2531991, rs2569888  rs2764264, rs2854152, rs28678024, rs28701981, rs301805, rs3116602, rs3184504, rs34517439, rs35624335  rs35696197, rs35874463, rs3625, rs3764002, rs3769885, rs3901421, rs4076427, rs4444637, rs4648620, rs465983  rs4752689, rs4752829, rs4776624, rs55745410, rs55872725, rs56363908, rs5742915, rs57904377, rs59000092  rs59950280, rs59985551, rs6054390, rs61729527, rs61838776, rs62621812, rs6567160, rs6860245, rs6910414  rs6977416, rs700677, rs7107356, rs7164187, rs72771070, rs72841270, rs73384223, rs7485647, rs75478182  rs7582516, rs76895963, rs777676, rs7816345, rs7858712, rs7952436, rs798528, rs8107967, rs894736, rs905938  rs9388490, rs9391254, rs963317, rs9647379, rs9659061, rs9807032, rs9853018, rs9905385, rs3814333, rs10483727  rs10748128, rs10779958, rs10883555, rs111365325, rs12051245, rs12055045, rs12099669, rs1211575, rs12509014  rs12679359, rs1319012, rs1355603, rs145147649, rs2035901, rs2174008, rs2209098, rs2229840, rs2270894  rs2539251, rs2663126, rs2721940, rs2812208, rs310796, rs33973388, rs35309034, rs3843750, rs3853252, rs41271299  rs41311445, rs42039, rs4244809, rs45528934, rs4909912, rs501250, rs55877758, rs6570509, rs68083605, rs6821305  rs6975015, rs704660 | |
| Usual walking pace | BMI/ body fat percentage/ type-2 diabetes /obesity | rs10828258, rs11039324, rs11881338, rs12042959, rs12461902, rs12883788, rs13107325, rs205262, rs2280406  rs2439823, rs2644135, rs273512, rs4516268, rs4643373, rs4715208, rs57800857, rs62048402 | |
| Hip OA | type-2 diabetes/ hyperthyroidism/ hypothyroidism | rs2785988, rs2929451, rs10896015 | |
| Knee OA | type-2 diabetes/ hyperthyroidism/ hypothyroidism | rs9277552, rs6499244 | |

ALM: appendicular lean mass; IVs: instrumental variables
