# Supplementary material for: Causal association between sarcopenia-related traits and osteoarthritis: A bidirectional 2-sample Mendelian randomization Study
Source: Medicine (Baltimore). 2025 Jul 11;104(28):e43069. doi: 10.1097/MD.0000000000043069 (PMC12263018; doi:10.1097/MD.0000000000043069)
Supplement: Supplementary file 2 [file medi-104-e43069-s002.docx]

**Supplementary Table 2. Detailed information of independent IVs for low grip strength, ALM and usual walking pace**

| Exposure | SNP | EA | OA | eaf.exposure | beta.exposure | se.exposure | pval.exposure | mr_keep | R2 | F-statistics |
| --- | --- | --- | --- | --- | --- | --- | --- | --- | --- | --- |
| low‐grip strength  low‐grip strength  low‐grip strength  low‐grip strength  low‐grip strength  low‐grip strength  low‐grip strength  low‐grip strength  low‐grip strength  low‐grip strength  low‐grip strength  low‐grip strength  ALM  ALM  ALM  ALM  ALM  ALM  ALM  ALM  ALM  ALM  ALM  ALM  ALM  ALM  ALM  ALM  ALM  ALM  ALM  ALM  ALM  ALM  ALM  ALM  ALM  ALM  ALM  ALM  ALM  ALM  ALM  ALM  ALM  ALM  ALM  ALM  ALM  ALM  ALM  ALM  ALM  ALM  ALM  ALM  ALM  ALM  ALM  ALM  ALM  ALM  ALM  ALM  ALM  ALM  ALM  ALM  ALM  ALM  ALM  ALM  ALM  ALM  ALM  ALM  ALM  ALM  ALM  ALM  ALM  ALM  ALM  ALM  ALM  ALM  ALM  ALM  ALM  ALM  ALM  ALM  ALM  ALM  ALM  ALM  ALM  ALM  ALM  ALM  ALM  ALM  ALM  ALM  ALM  ALM  ALM  ALM  ALM  ALM  ALM  ALM  ALM  ALM  ALM  ALM  ALM  ALM  ALM  ALM  ALM  ALM  ALM  ALM  ALM  ALM  ALM  ALM  ALM  ALM  ALM  ALM  ALM  ALM  ALM  ALM  ALM  ALM  ALM  ALM  ALM  ALM  ALM  ALM  ALM  ALM  ALM  ALM  ALM  ALM  ALM  ALM  ALM  ALM  ALM  ALM  ALM  ALM  ALM  ALM  ALM  ALM  ALM  ALM  ALM  ALM  ALM  ALM  ALM  ALM  ALM  ALM  ALM  ALM  ALM  ALM  ALM  ALM  ALM  ALM  ALM  ALM  ALM  ALM  ALM  ALM  ALM  ALM  ALM  ALM  ALM  ALM  ALM  ALM  ALM  ALM  ALM  ALM  ALM  ALM  ALM  ALM  ALM  ALM  ALM  ALM  ALM  ALM  ALM  ALM  ALM  ALM  ALM  ALM  ALM  ALM  ALM  ALM  ALM  ALM  ALM  ALM  ALM  ALM  ALM  ALM  ALM  ALM  ALM  ALM  ALM  ALM  ALM  ALM  ALM  ALM  ALM  ALM  ALM  ALM  ALM  ALM  ALM  ALM  ALM  ALM  ALM  ALM  ALM  ALM  ALM  ALM  ALM  ALM  ALM  ALM  ALM  ALM  ALM  ALM  ALM  ALM  ALM  ALM  ALM  ALM  ALM  ALM  ALM  ALM  ALM  ALM  ALM  ALM  ALM  ALM  ALM  ALM  ALM  ALM  ALM  ALM  ALM  ALM  ALM  ALM  ALM  ALM  ALM  ALM  ALM  ALM  ALM  ALM  ALM  ALM  ALM  ALM  ALM  ALM  ALM  ALM  ALM  ALM  ALM  ALM  ALM  ALM  ALM  ALM  ALM  ALM  ALM  ALM  ALM  ALM  ALM  ALM  ALM  ALM  ALM  ALM  ALM  ALM  ALM  ALM  ALM  ALM  ALM  ALM  ALM  ALM  ALM  ALM  ALM  ALM  ALM  ALM  ALM  ALM  ALM  ALM  ALM  ALM  ALM  ALM  ALM  ALM  ALM  ALM  ALM  ALM  ALM  ALM  ALM  ALM  ALM  ALM  ALM  ALM  ALM  ALM  ALM  ALM  ALM  ALM  ALM  ALM  ALM  ALM  ALM  ALM  ALM  ALM  ALM  ALM  ALM  ALM  ALM  ALM  ALM  ALM  ALM  ALM  ALM  ALM  ALM  ALM  ALM  ALM  ALM  ALM  ALM  ALM  ALM  ALM  ALM  ALM  ALM  ALM  ALM  ALM  ALM  ALM  ALM  ALM  ALM  ALM  ALM  ALM  ALM  ALM  ALM  ALM  ALM  ALM  ALM  ALM  ALM  ALM  ALM  ALM  ALM  ALM  ALM  ALM  ALM  ALM  ALM  ALM  ALM  ALM  ALM  ALM  ALM  ALM  ALM  ALM  ALM  ALM  ALM  ALM  ALM  ALM  ALM  ALM  ALM  ALM  ALM  ALM  ALM  ALM  ALM  ALM  ALM  ALM  ALM  ALM  ALM  ALM  ALM  ALM  ALM  ALM  ALM  ALM  ALM  ALM  ALM  ALM  ALM  ALM  ALM  ALM  ALM  ALM  ALM  ALM  ALM  ALM  ALM  ALM  ALM  ALM  ALM  ALM  ALM  ALM  ALM  ALM  ALM  ALM  ALM  ALM  ALM  ALM  ALM  ALM  ALM  ALM  ALM  ALM  ALM  ALM  ALM  ALM  ALM  ALM  ALM  ALM  ALM  ALM  ALM  ALM  ALM  ALM  ALM  ALM  ALM  ALM  ALM  ALM  ALM  ALM  ALM  ALM  ALM  ALM  Usual walking pace  Usual walking pace  Usual walking pace  Usual walking pace  Usual walking pace  Usual walking pace  Usual walking pace  Usual walking pace  Usual walking pace  Usual walking pace  Usual walking pace  Usual walking pace  Usual walking pace  Usual walking pace  Usual walking pace  Usual walking pace  Usual walking pace  Usual walking pace  Usual walking pace  Usual walking pace  Usual walking pace  Usual walking pace  Usual walking pace  Usual walking pace  Usual walking pace  Usual walking pace  Usual walking pace  Usual walking pace  Usual walking pace  Usual walking pace  Usual walking pace  Usual walking pace  Usual walking pace  Usual walking pace  Usual walking pace  Usual walking pace  Usual walking pace  Usual walking pace  Usual walking pace  Usual walking pace | rs12140813  rs11236213  rs34464763  rs2899611  rs143459567  rs8061064  rs62102286  rs79723785  rs958685  rs143384  rs7624084  rs185320691  rs12334478  rs13170063  rs7543136  rs73490624  rs2629448  rs11243202  rs56207248  rs11210229  rs200776140  rs2549677  rs35073631  rs34345560  rs112521375  rs6450136  rs7418410  rs113852999  rs7448554  rs7761910  rs6789000  rs73040028  rs13321258  rs2900208  rs7095087  rs947800  rs143384  rs6501381  rs6960741  rs3830008  rs76488803  rs139921635  rs2153091  rs8077636  rs6693481  rs10832963  rs73186333  rs9809116  rs2823990  rs4076108  rs11633371  rs113289555  rs6028716  rs8018486  rs2289976  rs28468602  rs9957318  rs2208404  rs115233595  rs4287835  rs3792819  rs111925803  rs1527149  rs9634212  rs7574162  rs201764844  rs395980  rs4815952  rs10922476  rs591668  rs17197114  rs17138358  rs2296316  rs4615815  rs11021305  rs12347137  rs62048221  rs13209685  rs6000890  rs1056747  rs11049704  rs34287  rs1290786  rs718603  rs9838614  rs2125125  rs10112506  rs11132166  rs80280630  rs61397287  rs113146332  rs59951000  rs4985445  rs73382475  rs45474992  rs11689546  rs548466113  rs9910161  rs9479012  rs7320878  rs8019890  rs35748083  rs501811  rs1035583  rs4733775  rs1216743  rs6943386  rs12622189  rs9525326  rs112873218  rs6066122  rs249681  rs680882  rs11991823  rs4383083  rs1436164  rs78444298  rs10421750  rs28592876  rs951366  rs10810474  rs12351226  rs61732778  rs2347808  rs2648725  rs36048468  rs62143873  rs71519447  rs7137546  rs9861931  rs9610447  rs8112948  rs7781964  rs1324538  rs2324154  rs12909863  rs12662115  rs112373502  rs2506697  rs8042545  rs2140046  rs715440  rs7731023  rs62033029  rs10840399  rs41298373  rs71384617  rs28787734  rs1430157  rs35756741  rs10514518  rs12423821  rs7910211  rs181766  rs201570119  rs55758152  rs8064946  rs6461948  rs188617336  rs72809820  rs551473284  rs58738817  rs117203652  rs6082354  rs13316  rs2627702  rs34776209  rs7701233  rs13103161  rs2994329  rs10005035  rs4735761  rs2788213  rs2142644  rs670129  rs7863102  rs11927331  rs4652902  rs4997514  rs11198591  rs35268848  rs12739693  rs6854705  rs4934377  rs6852065  rs9784904  rs116339650  rs10776560  rs1473441  rs2974337  rs11721522  rs4852257  rs4121583  rs2348496  rs4979576  rs2303423  rs12907384  rs7225068  rs61940146  rs603486  rs7171129  rs11233117  rs72908840  rs2678898  rs2490302  rs577289  rs10815304  rs6543146  rs10058744  rs8017006  rs1809179  rs10764692  rs9636364  rs56309431  rs6762851  rs7136054  rs34522021  rs7768382  rs7941305  rs12612857  rs13193017  rs4847378  rs3742250  rs485554  rs10008637  rs2252031  rs10827415  rs9579402  rs1800504  rs7768973  rs11672848  rs6931421  rs35453327  rs6874142  rs114018835  rs3818416  rs4504126  rs1977337  rs7359097  rs9385002  rs13127468  rs1177765  rs2871865  rs705953  rs75702986  rs10736029  rs10176654  rs9288695  rs62501195  rs1014526  rs80142996  rs76364830  rs4976262  rs74048171  rs113107560  rs8099461  rs75022676  rs496783  rs114299654  rs7692387  rs73158215  rs10917335  rs945508  rs9590328  rs2152090  rs9344126  rs4799799  rs76520574  rs4554207  rs12655296  rs62449290  rs12212816  rs6544743  rs182798714  rs10427685  rs73197345  rs149697773  rs4472895  rs11720869  rs9894577  rs2098695  rs3103223  rs79680939  rs2298333  rs7633464  rs12483401  rs4682483  rs11178643  rs11605297  rs10128781  rs8054549  rs59725651  rs117068593  rs12371664  rs10749157  rs11777835  rs2305141  rs3115084  rs60328144  rs2925155  rs34786000  rs11651280  rs75100513  rs112537273  rs11191208  rs4622329  rs631312  rs11158820  rs4940874  rs11684531  rs10241451  rs60408354  rs6962887  rs7646501  rs2993531  rs55633823  rs7286917  rs9832919  rs778384  rs9376478  rs149094387  rs112369231  rs36012032  rs7259285  rs9492799  rs2212926  rs2965074  rs1063582  rs332116  rs1557341  rs7185244  rs921142  rs994533  rs7730092  rs612577  rs655113  rs144109601  rs7968719  rs7971536  rs6450961  rs9696116  rs670318  rs17428810  rs7666804  rs28529055  rs35816944  rs17713523  rs10209278  rs2019203  rs59753424  rs200238746  rs12340775  rs78030362  rs78378222  rs17478946  rs72829852  rs62305043  rs1040977  rs1805165  rs2280463  rs12595051  rs1325596  rs4360494  rs31196  rs2577318  rs10822117  rs12512942  rs16844417  rs11158997  rs78457529  rs113827862  rs28736838  rs704832  rs757042  rs2807339  rs10041978  rs35464459  rs1291114  rs56207600  rs7078507  rs12474969  rs2885697  rs261999  rs7095472  rs1899040  rs7014590  rs116008080  rs28817902  rs8176632  rs723149  rs143076454  rs12299065  rs200739311  rs9817452  rs190801170  rs7083556  rs9931073  rs17205463  rs56263064  rs77542162  rs35963161  rs56208656  rs396015  rs9274305  rs5763821  rs17681189  rs3844  rs35732917  rs11760961  rs12663031  rs2651472  rs61925210  rs4252548  rs56034226  rs141277904  rs10222594  rs78180894  rs7328187  rs4274112  rs66613683  rs6739394  rs2487  rs2607234  rs876122  rs10036789  rs4895801  rs17773965  rs117818446  rs2609334  rs13109280  rs7367519  rs6899155  rs11175919  rs75172776  rs28757154  rs1004982  rs4754296  rs10208668  rs6469845  rs1444628  rs2596144  rs12702693  rs563738408  rs9828525  rs113823725  rs1341215  rs7229520  rs4763327  rs7007389  rs2007022  rs72801818  rs73125634  rs7598430  rs372532055  rs985136  rs3103268  rs116493405  rs6844176  rs6963134  rs12713004  rs61944841  rs9513510  rs2126942  rs2304655  rs244711  rs7228151  rs13209574  rs2071518  rs320826  rs6505216  rs10170971  rs62103240  rs7428883  rs35892992  rs1405227  rs12037677  rs36226649  rs6054491  rs60804050  rs372748371  rs10283100  rs114192718  rs10040039  rs62372061  rs200801362  rs10099846  rs12344515  rs7129320  rs291970  rs6923230  rs853168  rs12700901  rs8084413  rs3003137  rs6738207  rs147110934  rs4619294  rs6919321  rs7893378  rs10880272  rs113232639  rs11014285  rs165849  rs7301341  rs57307236  rs377599  rs4282339  rs62177315  rs12185775  rs7512641  rs2138374  rs10940169  rs11590254  rs8180765  rs200439  rs8904  rs4849904  rs140440099  rs1447691  rs177592  rs62466110  rs1330826  rs212526  rs113671109  rs57287582  rs447352  rs2578565  rs76693355  rs2297600  rs113825410  rs11548200  rs12747822  rs55680124  rs1592  rs139398785  rs11682482  rs830627  rs6763292  rs28519617  rs11732213  rs144333966  rs35711462  rs11152989  rs4839898  rs9366651  rs7789719  rs11761141  rs7795394  rs4109292  rs7896518  rs10883618  rs9783304  rs10750025  rs10862220  rs2645979  rs2170670  rs45583845  rs8011870  rs8010773  rs11848096  rs8028757  rs11150623  rs11077815  rs613872  rs891387  rs2602731  rs2037735  rs819167 | T  A  A  T  T  A  T  T  A  A  T  C  C  A  T  T  T  T  T  A  A  A  T  A  A  A  T  A  A  T  T  T  A  A  A  C  A  T  T  A  A  T  C  T  T  T  A  A  A  A  T  T  A  A  A  T  A  A  T  T  A  T  C  A  T  T  T  T  A  A  T  C  T  A  T  A  T  T  T  A  C  A  T  T  T  T  A  T  T  A  C  T  A  T  T  A  T  A  A  A  A  T  T  A  A  A  T  A  A  T  C  C  T  A  A  T  A  T  A  T  T  T  A  A  A  T  A  A  A  C  T  A  A  A  A  C  A  T  A  A  T  A  A  A  T  A  T  T  T  T  T  T  T  T  T  A  C  T  T  T  T  A  A  A  A  T  T  T  A  T  C  A  A  A  T  A  A  A  C  A  A  C  T  A  T  T  A  T  A  T  A  T  T  A  T  T  T  A  A  T  T  C  T  T  A  A  T  T  A  A  T  A  A  T  T  C  T  T  T  A  A  T  C  C  T  T  T  T  T  A  T  T  A  T  T  A  A  T  T  A  A  T  C  A  A  T  T  C  A  T  C  A  T  A  T  A  A  A  T  A  A  A  T  A  C  T  A  T  T  T  A  T  T  A  C  A  A  A  A  A  A  T  A  T  A  T  A  A  A  T  A  A  T  A  T  T  A  T  A  T  T  T  T  T  A  A  A  A  A  A  T  A  T  A  A  T  A  A  A  A  C  T  A  A  A  A  T  T  T  A  T  T  C  T  T  T  A  C  A  T  T  T  T  T  T  A  A  T  A  A  A  A  A  T  A  T  T  T  A  A  A  A  C  A  A  A  A  A  C  T  T  T  A  T  T  A  T  C  A  A  A  T  T  A  T  T  A  A  T  A  A  A  T  T  A  A  C  T  C  A  A  A  A  T  A  A  A  T  C  T  T  A  T  T  T  A  C  T  A  T  T  T  A  A  C  C  T  A  T  A  T  T  A  A  A  T  T  C  T  T  T  T  A  T  C  A  A  T  A  A  T  T  T  A  C  T  A  T  A  A  A  C  T  T  T  T  T  T  C  T  C  A  A  T  A  T  T  C  A  C  A  T  A  T  T  T  T  A  T  T  C  A  A  T  A  T  C  A  A  T  A  A  A  T  A  T  A  T  C  A  T  C  A  A  A  A  C  A  A  C  T  C  T  T  T  T  T  T  G  G  C  A  T  C  G  G  A  G  G  C  G  G  T  A  T  C  G  A  A  G  A  T  T  G  A  A  G  A  C  C  T  T  C  T  C  G  T  G | C  G  T  G  C  T  G  C  C  G  C  G  G  G  C  C  C  C  C  G  G  G  C  G  T  C  C  G  C  C  G  C  G  C  G  G  G  C  C  C  G  G  G  G  C  G  C  G  G  T  G  G  G  G  G  G  G  G  C  C  G  C  G  C  C  C  G  C  G  G  C  G  C  G  C  C  C  G  C  G  G  G  C  C  G  C  G  C  C  T  G  C  G  C  C  G  C  G  G  G  C  C  C  G  G  G  C  G  G  C  G  G  G  G  G  C  G  G  G  C  C  C  G  G  T  C  G  G  T  G  C  T  G  T  C  G  G  C  G  G  C  T  G  G  C  G  C  C  C  C  C  C  C  C  C  G  G  C  C  C  C  G  G  C  C  C  C  C  T  C  G  C  G  C  C  T  C  C  G  G  C  G  C  T  C  C  G  C  G  C  G  G  C  G  C  C  C  G  G  C  C  G  G  C  T  T  C  G  G  G  C  C  G  G  C  G  C  C  C  G  G  G  G  G  C  C  G  C  C  T  C  G  C  G  C  C  C  C  C  T  C  C  G  G  G  G  C  G  C  C  G  G  C  C  G  G  G  G  C  G  G  G  C  G  G  C  C  C  C  C  G  C  G  T  G  T  G  T  G  G  G  C  G  C  G  C  G  T  G  C  C  G  C  G  C  C  G  C  C  C  G  G  C  C  G  G  G  G  G  G  C  G  G  G  C  C  G  G  G  G  G  C  C  G  G  C  G  G  C  C  C  C  G  G  C  C  C  G  T  C  C  C  C  C  G  G  G  C  C  G  G  G  G  G  G  C  C  C  C  G  G  G  G  C  T  G  G  G  G  C  C  C  C  C  C  G  C  G  G  G  G  G  C  G  C  C  G  G  C  G  G  G  C  G  G  T  G  C  G  G  G  G  T  C  C  C  C  C  G  C  G  T  C  C  C  G  G  G  G  C  C  C  G  G  G  G  C  G  C  G  C  C  G  G  G  C  G  G  C  C  C  C  G  C  G  G  G  C  T  C  C  G  C  C  G  C  G  C  G  G  G  G  C  C  C  C  G  C  G  G  G  G  G  C  G  C  C  G  G  G  G  C  G  C  C  C  C  G  C  C  G  C  G  G  G  G  G  G  G  G  G  G  G  C  G  C  G  C  G  T  C  G  T  G  C  G  G  G  G  G  C  G  C  C  G  C  C  C  T  A  T  T  C  A  C  T  G  A  T  T  A  A  C  G  G  T  T  T  G  A  G  G  C  T  G  G  C  G  T  T  A  G  T  G  T  A  C  A | 0.1856  0.3136  0.3943  0.4951  0.0386  0.4627  0.5641  0.9835  0.5142  0.5912  0.5577  0.1047  0.5036  0.5921  0.7209  0.2002  0.9379  0.514  0.0655  0.3832  0.1474  0.9006  0.5669  0.1952  0.8659  0.6913  0.4089  0.1688  0.4999  0.3086  0.6449  0.2292  0.2301  0.354  0.3529  0.0357  0.5962  0.8867  0.8831  0.7964  0.0826  0.0236  0.6995  0.4176  0.3049  0.2555  0.976  0.5919  0.6773  0.7551  0.4764  0.2333  0.2587  0.8086  0.3054  0.5138  0.652  0.5842  0.9376  0.466  0.9146  0.2851  0.5933  0.221  0.757  0.6903  0.7365  0.4779  0.4432  0.396  0.8228  0.3982  0.5357  0.6031  0.6164  0.7977  0.2307  0.1594  0.6339  0.5883  0.707  0.341  0.4307  0.2774  0.6119  0.217  0.6102  0.9638  0.1115  0.9253  0.0381  0.0419  0.5435  0.089  0.0362  0.5599  0.2725  0.7148  0.9056  0.6034  0.5313  0.5682  0.886  0.6177  0.364  0.7193  0.5998  0.7423  0.8125  0.1054  0.2369  0.6175  0.2394  0.3206  0.6466  0.5572  0.0196  0.312  0.2051  0.6068  0.4177  0.1712  0.071  0.5139  0.2134  0.2088  0.5033  0.1102  0.5751  0.6035  0.7471  0.2856  0.1854  0.3836  0.5096  0.2512  0.6734  0.8648  0.6805  0.2424  0.6366  0.4574  0.4251  0.2064  0.1013  0.1007  0.3457  0.0583  0.3117  0.0924  0.6855  0.8425  0.8413  0.678  0.7902  0.3263  0.1103  0.6732  0.2983  0.324  0.3649  0.2477  0.03  0.3325  0.4301  0.4811  0.2475  0.5717  0.3891  0.2017  0.7177  0.7143  0.7104  0.672  0.4135  0.5526  0.3305  0.7683  0.8408  0.3684  0.0119  0.4683  0.2022  0.7297  0.5768  0.8454  0.873  0.4995  0.2764  0.5002  0.5875  0.4237  0.6192  0.5129  0.8814  0.8905  0.4677  0.4094  0.3638  0.3827  0.32  0.5465  0.0491  0.6198  0.9137  0.7192  0.7944  0.4421  0.5027  0.6725  0.1584  0.363  0.5429  0.1164  0.6427  0.5178  0.4547  0.5229  0.6867  0.2382  0.1369  0.6121  0.5377  0.3154  0.538  0.1541  0.7392  0.9216  0.5523  0.411  0.5246  0.6776  0.075  0.8862  0.0359  0.2355  0.9725  0.1489  0.6037  0.7606  0.4629  0.5319  0.8841  0.3353  0.1861  0.91  0.7815  0.7788  0.8288  0.3946  0.9307  0.0634  0.6844  0.2583  0.5882  0.9445  0.2079  0.5352  0.9641  0.1854  0.7745  0.6699  0.4636  0.8583  0.4398  0.4863  0.3568  0.0413  0.4854  0.6251  0.1592  0.5128  0.1909  0.9726  0.9358  0.1367  0.9554  0.7568  0.6686  0.318  0.3089  0.2603  0.0449  0.5677  0.4786  0.9784  0.1534  0.6407  0.2328  0.7142  0.4486  0.6607  0.1895  0.7276  0.6416  0.5267  0.4039  0.8834  0.4685  0.2612  0.5539  0.0605  0.9144  0.7701  0.2057  0.3496  0.7233  0.3128  0.1881  0.8672  0.7912  0.0732  0.6928  0.7365  0.4106  0.2518  0.2539  0.6426  0.2226  0.2348  0.9187  0.735  0.091  0.4435  0.1574  0.2106  0.4521  0.2351  0.2804  0.3326  0.2241  0.6021  0.3158  0.1918  0.8511  0.6994  0.0418  0.4714  0.4945  0.6772  0.6818  0.0485  0.6964  0.6111  0.4368  0.0066  0.4066  0.6757  0.4909  0.2666  0.3939  0.0552  0.9253  0.9877  0.6996  0.0621  0.9362  0.1662  0.7204  0.6952  0.2801  0.5476  0.5541  0.5724  0.7488  0.763  0.6376  0.1238  0.193  0.0117  0.9392  0.3017  0.2509  0.276  0.2414  0.5173  0.8842  0.8903  0.111  0.3908  0.3006  0.665  0.3702  0.4665  0.7962  0.7386  0.0238  0.8824  0.1308  0.4372  0.0186  0.1274  0.3812  0.3876  0.926  0.5393  0.4721  0.4477  0.7142  0.9774  0.4757  0.1103  0.4661  0.2052  0.6093  0.4231  0.752  0.7157  0.6332  0.4296  0.5117  0.8953  0.0218  0.1409  0.0228  0.3226  0.074  0.5024  0.6267  0.8302  0.3689  0.5644  0.0524  0.1212  0.5391  0.5532  0.1412  0.0202  0.7741  0.3382  0.3154  0.5518  0.0262  0.9344  0.1426  0.6163  0.8536  0.035  0.2391  0.6904  0.1304  0.4542  0.0339  0.4113  0.54  0.1374  0.6616  0.6984  0.3727  0.2315  0.3005  0.2782  0.5046  0.3664  0.5105  0.9018  0.0537  0.5446  0.3476  0.2746  0.4138  0.6988  0.4202  0.5682  0.686  0.794  0.1  0.264  0.4954  0.233  0.5022  0.071  0.2192  0.2087  0.3167  0.2825  0.9333  0.7596  0.2559  0.9351  0.0555  0.0748  0.6133  0.0979  0.8397  0.6684  0.2402  0.1661  0.2291  0.4657  0.2473  0.4055  0.4693  0.4283  0.4007  0.0243  0.6947  0.6075  0.1143  0.6161  0.493  0.1654  0.6975  0.6732  0.6016  0.383  0.2075  0.0956  0.1172  0.7405  0.304  0.3816  0.6886  0.7826  0.7793  0.363  0.6203  0.0234  0.3555  0.1013  0.933  0.2268  0.3993  0.78  0.1878  0.1408  0.6575  0.8838  0.17194  0.224386  0.066034  0.09792  0.154913  0.395455  0.140415  0.680801  0.417471  0.780939  0.269813  0.195605  0.014646  0.511213  0.312264  0.109786  0.507037  0.782126  0.325697  0.624965  0.493943  0.427878  0.372248  0.689164  0.681757  0.674897  0.357204  0.606925  0.034279  0.288257  0.38208  0.386669  0.130387  0.644857  0.623657  0.825829  0.49494  0.681058  0.120628  0.936466 | 0.0511  -0.0504  0.0544  -0.0431  0.1185  0.0407  0.0487  -0.1674  -0.0428  0.0545  0.0428  0.0913  0.0161  -0.0152  -0.021  -0.0184  -0.0368  -0.0302  -0.0273  -0.0125  -0.015  0.0392  -0.0112  0.0219  0.0202  -0.0201  0.0155  0.0246  -0.0132  -0.016  0.0121  0.0168  0.0133  0.0255  0.0117  -0.0331  -0.0725  0.0341  -0.0185  0.0154  -0.024  0.0385  -0.0115  -0.0192  0.0143  0.0203  -0.0373  0.016  -0.0127  -0.0174  0.0216  -0.0206  -0.021  0.0138  0.0144  -0.0112  -0.0187  0.0111  0.0264  -0.0147  -0.021  0.0122  -0.0113  0.0471  0.0153  -0.0196  0.0184  0.0161  -0.016  -0.0174  -0.0177  0.0157  0.0192  0.0246  -0.0152  0.046  -0.0242  0.0277  0.0135  0.0155  0.0183  0.0187  -0.0143  0.0131  0.0185  -0.0158  0.012  0.0292  -0.0168  -0.0235  0.0311  -0.04  0.0175  0.0254  -0.0617  0.0238  -0.036  -0.0162  -0.0263  -0.015  0.025  -0.0197  0.0199  0.0148  0.0143  -0.0152  -0.0106  0.0272  0.0184  0.0216  -0.0127  -0.0117  -0.0133  -0.0161  0.0111  -0.0139  -0.0465  -0.0145  0.03  -0.0205  0.0143  0.0218  0.023  -0.0125  0.0165  0.0254  -0.0115  -0.0716  -0.0142  0.011  0.0152  -0.0297  0.0261  0.0237  0.015  0.0189  0.0152  -0.0179  0.0141  0.0287  0.0192  -0.017  -0.0166  -0.0141  -0.0209  0.0185  0.0133  0.0385  0.0182  -0.0378  0.0171  -0.0161  -0.0175  -0.0221  -0.0194  0.0145  -0.0195  0.0112  0.0138  -0.0111  0.017  -0.0257  -0.0346  0.024  0.0115  0.0191  -0.0317  0.0179  -0.0284  0.0155  0.0175  -0.0331  0.0123  -0.0181  -0.0118  0.011  -0.0147  0.0129  0.0231  0.0148  0.0737  -0.0145  0.0173  -0.0172  0.0131  0.0217  0.0175  -0.0157  0.0198  0.0117  -0.0106  0.0231  0.0118  0.0136  0.0317  -0.0168  0.0269  -0.0141  -0.0112  -0.0127  0.012  0.0176  0.037  0.0129  0.0221  0.0125  -0.0151  -0.0154  0.0146  -0.0122  -0.0155  0.0111  0.011  0.0167  0.0218  -0.0497  0.0126  0.0201  0.0129  -0.0124  0.0164  0.0136  0.0136  0.0342  0.013  0.0176  0.0142  -0.0203  -0.0125  -0.024  -0.0171  0.0279  0.0283  -0.0288  0.0303  -0.0279  -0.046  -0.0186  0.0121  0.0147  -0.0123  0.0232  0.0493  0.019  -0.0163  -0.0193  0.0128  0.0127  0.0198  0.0138  -0.0255  -0.0471  0.0245  -0.0121  0.019  0.0246  -0.0163  0.0124  0.0304  0.0169  0.0163  0.0198  0.0128  -0.0153  -0.0118  0.0185  -0.0113  -0.0449  0.0114  -0.011  0.0213  0.0162  0.0217  -0.0376  -0.0269  0.0211  0.0262  -0.0148  0.0141  -0.031  0.0255  -0.0126  0.029  -0.0267  0.0175  0.0387  -0.0165  -0.0109  0.0146  -0.0172  -0.0251  0.0167  0.0403  0.0168  -0.0113  0.0152  -0.0183  0.024  0.0144  -0.015  -0.015  0.0267  -0.0199  0.0212  0.0147  0.0149  -0.0126  0.0235  -0.0148  0.0172  -0.0145  0.0259  0.0127  0.017  -0.0176  0.0146  -0.0171  0.0179  0.0283  -0.0186  0.0366  -0.0181  0.0298  -0.0132  0.0182  -0.022  0.0125  0.0185  -0.0206  -0.0153  0.0148  0.0111  -0.0313  0.0131  0.0152  -0.0188  -0.0278  0.0134  -0.0194  0.0118  0.0188  -0.0413  0.0155  0.0167  -0.0147  -0.1088  0.0114  0.0141  0.0189  0.02  -0.0155  -0.0287  -0.0216  -0.138  0.0192  0.0309  -0.0269  -0.024  -0.0184  0.0147  0.0179  0.0287  -0.0198  -0.0107  -0.0139  0.0176  -0.0162  0.0226  0.0131  -0.0904  0.0235  -0.0117  -0.012  0.014  -0.0162  -0.0172  0.0342  0.0173  0.0192  0.02  0.0167  -0.0323  0.0175  -0.0267  0.0152  0.0228  -0.0415  -0.023  -0.0158  0.0276  -0.0499  -0.024  0.0128  0.0165  0.0286  -0.0109  -0.0151  -0.0263  0.015  -0.0576  -0.0157  -0.0284  -0.0178  0.0298  -0.0191  -0.0131  0.0132  -0.0204  -0.0133  -0.0195  0.0108  -0.0187  -0.0753  0.0226  0.0384  0.0115  0.0338  -0.0116  0.0217  -0.015  -0.0144  -0.014  0.0302  -0.0162  -0.0163  -0.0149  -0.0163  0.0423  0.0171  -0.0131  -0.0164  0.0282  0.0349  -0.0232  -0.0193  0.0117  0.0167  0.0415  0.0135  0.024  0.0223  0.0173  -0.0587  0.0121  -0.0259  0.0229  -0.0224  0.0123  -0.0134  0.0179  0.0313  -0.0195  -0.016  0.0175  -0.0138  0.0291  0.0287  -0.0129  -0.0137  -0.0367  0.0253  -0.0181  0.0127  -0.0113  0.0279  0.0185  -0.0292  -0.0236  0.0154  -0.0498  -0.0157  0.0212  -0.0277  -0.0182  0.0129  0.0178  -0.0485  0.0142  -0.0217  -0.0316  -0.0575  -0.0215  0.0163  0.0394  -0.0405  0.0116  -0.0163  -0.0389  0.024  0.0127  0.0156  -0.0184  -0.0127  -0.0114  0.0127  -0.0722  -0.0144  0.0192  0.0175  0.0138  0.0327  0.0342  0.0157  0.0255  -0.0155  0.0217  -0.0311  0.0177  -0.0167  -0.0185  0.0149  -0.011  -0.0186  0.0151  0.0128  -0.0157  -0.0111  0.0613  0.0181  -0.022  0.0371  0.0162  -0.0214  0.015  0.0159  -0.0181  -0.0141  0.0268  -0.0115607  -0.00881182  -0.016041  0.0117683  -0.010742  0.0074513  -0.00999513  0.00801894  0.00742054  0.00958833  -0.00812933  0.0091614  0.0304614  -0.0071659  -0.00751226  0.0130564  0.00948542  0.00854199  -0.00789936  0.00930493  0.00735336  0.00999095  0.0078103  0.00763598  -0.0083554  0.00840767  0.00861798  -0.00708769  -0.0198998  -0.00782739  -0.00816754  -0.00750831  0.0107284  -0.00968813  -0.00713336  -0.0147074  0.00786028  -0.00766675  -0.0112677  -0.0157993 | 0.0094  0.008  0.0086  0.0074  0.0189  0.0074  0.0074  0.0293  0.0074  0.0075  0.0074  0.0146  0.0019  0.0019  0.0021  0.0024  0.004  0.0019  0.0038  0.0019  0.0027  0.0032  0.0019  0.0024  0.0028  0.002  0.0019  0.0025  0.002  0.002  0.002  0.0022  0.0022  0.002  0.002  0.0051  0.0019  0.003  0.0029  0.0023  0.0034  0.0062  0.0021  0.0019  0.002  0.0022  0.0063  0.0019  0.002  0.0022  0.0019  0.0023  0.0022  0.0024  0.002  0.0019  0.002  0.0019  0.0039  0.0019  0.0034  0.0021  0.0019  0.0023  0.0022  0.0021  0.0021  0.0019  0.0019  0.0019  0.0025  0.0019  0.0019  0.0019  0.0019  0.0024  0.0022  0.0026  0.002  0.0019  0.0021  0.002  0.0019  0.0021  0.0019  0.0023  0.0019  0.005  0.003  0.0036  0.0049  0.0047  0.0019  0.0033  0.0051  0.0019  0.0021  0.0021  0.0032  0.0019  0.0019  0.0019  0.003  0.0019  0.002  0.0021  0.0019  0.0021  0.0024  0.0031  0.0023  0.0019  0.0022  0.002  0.002  0.0019  0.0068  0.0021  0.0023  0.0019  0.0019  0.0025  0.0037  0.0019  0.0023  0.0023  0.0019  0.003  0.0019  0.0019  0.0022  0.0022  0.0024  0.0019  0.0019  0.0022  0.002  0.0028  0.002  0.0022  0.0019  0.0019  0.0019  0.0023  0.0031  0.0031  0.0021  0.0041  0.002  0.0033  0.002  0.0027  0.0026  0.002  0.0023  0.002  0.003  0.002  0.0021  0.002  0.002  0.0022  0.0055  0.002  0.0019  0.0019  0.0022  0.0019  0.0019  0.0023  0.0021  0.0021  0.0021  0.002  0.0019  0.0019  0.002  0.0022  0.0027  0.002  0.0101  0.0019  0.0024  0.0021  0.0019  0.0026  0.0029  0.0019  0.0021  0.0019  0.0019  0.0019  0.002  0.0019  0.0029  0.003  0.0019  0.0019  0.002  0.002  0.002  0.0019  0.0043  0.0019  0.0034  0.0021  0.0023  0.0019  0.0019  0.002  0.0026  0.002  0.0019  0.0029  0.002  0.0019  0.0019  0.0019  0.0021  0.0022  0.0027  0.0019  0.0019  0.002  0.0019  0.0026  0.0021  0.0035  0.0019  0.0019  0.0019  0.002  0.0036  0.0031  0.0051  0.0022  0.0058  0.0026  0.0019  0.0022  0.0019  0.0019  0.003  0.002  0.0025  0.0035  0.0023  0.0023  0.0025  0.002  0.0037  0.0039  0.002  0.0022  0.0019  0.0041  0.0023  0.0019  0.0051  0.0024  0.0023  0.002  0.0019  0.0027  0.0019  0.0019  0.002  0.0049  0.0019  0.002  0.0026  0.0019  0.0024  0.0062  0.0039  0.0028  0.0047  0.0022  0.002  0.002  0.002  0.0022  0.0046  0.0019  0.0019  0.0067  0.0026  0.002  0.0022  0.0021  0.0019  0.002  0.0024  0.0021  0.002  0.0019  0.0019  0.0031  0.0019  0.0022  0.0019  0.004  0.0034  0.0022  0.0024  0.002  0.0021  0.0021  0.0024  0.0028  0.0023  0.0036  0.0021  0.0021  0.0019  0.0022  0.0023  0.002  0.0022  0.0022  0.0034  0.0021  0.0033  0.0019  0.0026  0.0023  0.0019  0.0022  0.0021  0.002  0.0023  0.0019  0.002  0.0024  0.0026  0.0021  0.0048  0.0019  0.0019  0.002  0.002  0.0044  0.002  0.0019  0.0019  0.0117  0.0019  0.002  0.0019  0.0022  0.0023  0.0043  0.0036  0.0087  0.0021  0.0039  0.004  0.0025  0.0021  0.0021  0.0021  0.0019  0.0019  0.0019  0.0022  0.0022  0.002  0.0029  0.0024  0.0088  0.004  0.002  0.0022  0.0021  0.0022  0.0019  0.0029  0.0031  0.003  0.0019  0.002  0.002  0.0019  0.0019  0.0023  0.0022  0.0063  0.0029  0.0028  0.0019  0.007  0.0028  0.002  0.0019  0.0036  0.0019  0.0019  0.0019  0.0021  0.0064  0.0019  0.0034  0.0019  0.0025  0.002  0.0019  0.0022  0.0021  0.002  0.0019  0.0019  0.0031  0.0065  0.0027  0.0069  0.002  0.0037  0.0019  0.002  0.0025  0.0019  0.0019  0.0043  0.0029  0.0019  0.0019  0.0027  0.0068  0.0022  0.002  0.002  0.0019  0.0059  0.0038  0.0027  0.002  0.0027  0.0051  0.0022  0.002  0.0028  0.0019  0.0053  0.0019  0.0019  0.0027  0.002  0.0021  0.002  0.0022  0.0021  0.0021  0.0019  0.002  0.002  0.0031  0.0042  0.0019  0.002  0.0021  0.002  0.0021  0.0019  0.0019  0.0022  0.0023  0.0032  0.0021  0.0022  0.0023  0.0019  0.0037  0.0023  0.0023  0.002  0.0021  0.0038  0.0022  0.0021  0.0039  0.0041  0.0036  0.0019  0.0032  0.0035  0.002  0.0022  0.0025  0.0022  0.0019  0.0022  0.0019  0.0019  0.0019  0.0019  0.0062  0.0021  0.0019  0.0031  0.0019  0.0019  0.0026  0.0021  0.002  0.0019  0.0019  0.0023  0.0032  0.003  0.0022  0.002  0.0019  0.002  0.0023  0.0023  0.002  0.0019  0.0063  0.002  0.0031  0.0041  0.0023  0.0019  0.0023  0.0025  0.0029  0.002  0.003  0.00168167  0.00151778  0.00255177  0.00213998  0.00174984  0.00128817  0.00182329  0.00135595  0.00128505  0.00153076  0.0014336  0.00159593  0.00544615  0.00126947  0.00136699  0.00206037  0.00127132  0.00153719  0.00135132  0.00130867  0.00126787  0.00129269  0.00130834  0.00136834  0.00136528  0.00135051  0.00132024  0.0012972  0.0036328  0.00140635  0.00130079  0.00130696  0.00192335  0.0013224  0.00130691  0.00167052  0.00126616  0.00136907  0.00194308  0.00259282 | 5.44E-08  2.98E-10  2.52E-10  5.73E-09  3.61E-10  3.80E-08  4.67E-11  1.11E-08  7.30E-09  3.68E-13  7.30E-09  4.02E-10  2.38E-17  1.24E-15  1.52E-23  1.77E-14  3.58E-20  6.89E-57  6.76E-13  4.74E-11  2.77E-08  1.68E-34  3.75E-09  7.17E-20  5.42E-13  9.19E-24  3.41E-16  7.57E-23  4.11E-11  1.24E-15  1.45E-09  2.23E-14  1.49E-09  3.12E-37  4.92E-09  8.57E-11  0  6.13E-30  1.78E-10  2.15E-11  1.68E-12  5.31E-10  4.35E-08  5.24E-24  8.68E-13  2.78E-20  3.21E-09  3.73E-17  2.15E-10  2.59E-15  6.01E-30  3.35E-19  1.36E-21  8.92E-09  6.02E-13  3.75E-09  8.76E-21  5.15E-09  1.29E-11  1.02E-14  6.56E-10  6.27E-09  2.72E-09  3.36E-93  3.54E-12  1.03E-20  1.92E-18  2.38E-17  3.73E-17  5.29E-20  1.44E-12  1.42E-16  5.24E-24  2.43E-38  1.24E-15  7.03E-82  3.82E-28  1.67E-26  1.48E-11  3.41E-16  2.93E-18  8.76E-21  5.22E-14  4.43E-10  2.10E-22  6.44E-12  2.69E-10  5.22E-09  2.14E-08  6.68E-11  2.20E-10  1.73E-17  3.25E-20  1.39E-14  1.08E-33  5.36E-36  7.11E-66  1.22E-14  2.06E-16  2.91E-15  1.53E-39  3.45E-25  3.28E-11  6.73E-15  8.68E-13  4.55E-13  2.42E-08  2.28E-38  1.77E-14  3.22E-12  3.36E-08  7.37E-10  1.49E-09  8.28E-16  2.86E-08  2.56E-13  8.02E-12  5.03E-12  6.92E-39  3.86E-27  5.22E-14  2.78E-18  5.09E-10  4.74E-11  7.29E-13  2.36E-28  1.42E-09  6.80E-126  7.80E-14  7.06E-09  4.88E-12  1.56E-41  1.52E-27  1.04E-35  2.91E-15  8.63E-18  2.96E-14  1.63E-10  1.79E-12  6.75E-39  5.24E-24  3.64E-19  2.40E-18  8.76E-10  1.56E-11  2.41E-09  2.40E-10  5.99E-21  9.03E-20  2.23E-30  1.23E-17  2.48E-09  1.69E-11  2.19E-28  3.32E-17  4.17E-13  8.03E-11  2.14E-08  4.98E-11  2.86E-08  1.90E-17  1.58E-31  3.16E-10  3.55E-33  1.42E-09  8.94E-24  4.54E-47  4.47E-21  1.62E-50  1.59E-11  7.86E-17  5.69E-56  4.71E-09  1.43E-19  5.28E-10  7.06E-09  1.98E-13  4.53E-09  1.17E-17  1.36E-13  2.94E-13  2.32E-14  5.66E-13  2.60E-16  5.40E-12  7.05E-17  1.59E-09  1.42E-16  4.16E-21  7.37E-10  2.42E-08  5.21E-34  3.64E-09  8.19E-13  8.19E-28  2.14E-08  1.67E-45  1.16E-13  2.14E-08  2.15E-10  1.97E-09  1.98E-20  7.65E-18  1.13E-11  8.03E-11  2.64E-09  5.20E-11  5.26E-16  1.54E-14  1.06E-09  2.50E-09  2.86E-08  7.06E-09  8.48E-09  1.15E-27  8.01E-151  3.32E-11  3.73E-26  8.11E-10  1.74E-08  1.25E-09  8.19E-13  8.19E-13  1.48E-65  7.80E-12  1.29E-11  1.36E-11  6.63E-09  4.74E-11  1.41E-36  2.26E-19  3.15E-44  3.81E-15  1.54E-20  2.83E-09  7.46E-37  2.17E-15  8.44E-13  1.91E-10  2.36E-11  9.56E-11  2.73E-34  1.10E-60  2.10E-21  7.03E-11  3.50E-08  2.62E-08  3.36E-08  2.38E-15  5.20E-12  5.51E-12  1.40E-33  1.68E-34  3.80E-08  1.52E-23  1.97E-09  1.37E-12  6.74E-11  2.51E-09  1.90E-12  1.37E-12  4.16E-23  1.62E-11  1.46E-08  5.28E-10  2.10E-22  1.60E-08  5.04E-20  1.97E-09  3.80E-08  2.56E-16  1.51E-17  1.54E-19  1.32E-09  5.29E-12  4.86E-14  2.48E-08  1.73E-11  1.79E-12  3.47E-54  3.12E-37  1.02E-08  2.89E-10  7.42E-45  3.25E-20  7.64E-09  2.21E-10  5.04E-08  3.22E-11  2.60E-16  7.63E-40  6.83E-17  2.81E-63  1.24E-15  1.60E-08  1.24E-15  5.88E-22  9.79E-15  3.48E-14  9.22E-12  2.91E-15  2.47E-11  4.83E-09  5.61E-22  9.07E-10  9.33E-14  1.97E-09  4.54E-29  6.97E-10  8.11E-10  2.89E-10  6.27E-13  1.47E-09  5.72E-16  1.98E-20  3.22E-11  1.05E-13  3.55E-19  7.21E-38  2.80E-17  5.05E-27  6.75E-18  1.71E-19  3.72E-12  2.56E-12  1.12E-21  4.74E-11  4.13E-17  1.02E-22  2.01E-14  1.24E-10  5.15E-09  3.32E-55  4.81E-08  5.03E-09  3.48E-19  6.97E-09  1.76E-12  1.78E-24  3.64E-09  5.46E-21  6.21E-21  9.19E-15  1.50E-18  1.02E-14  1.42E-20  1.97E-09  1.79E-12  2.59E-23  9.82E-20  1.59E-11  2.48E-11  1.97E-09  1.16E-56  6.08E-20  2.32E-15  1.76E-11  7.99E-22  1.92E-18  2.56E-12  1.54E-17  1.50E-51  1.99E-25  1.79E-08  2.65E-10  1.24E-15  5.50E-16  6.54E-15  4.81E-08  9.35E-25  4.23E-09  4.92E-09  4.91E-08  2.62E-11  1.79E-13  1.40E-19  4.24E-32  2.40E-08  1.55E-10  6.53E-26  6.83E-17  1.14E-58  3.25E-20  7.42E-45  3.88E-11  3.63E-25  4.48E-11  2.17E-15  1.67E-08  8.25E-48  1.01E-12  1.02E-17  1.55E-10  3.81E-18  1.95E-15  9.65E-09  1.91E-15  1.42E-43  9.14E-13  2.26E-19  1.42E-16  6.66E-17  7.36E-21  9.31E-33  1.30E-21  5.40E-12  1.97E-09  2.62E-22  2.93E-11  1.03E-24  1.31E-08  1.62E-09  4.93E-31  5.74E-17  2.62E-08  8.92E-09  6.53E-20  1.03E-09  1.99E-27  1.97E-09  3.48E-14  1.73E-13  2.17E-12  2.32E-08  9.57E-18  4.43E-15  1.57E-09  4.95E-10  7.68E-15  5.75E-11  2.40E-16  7.83E-50  3.31E-09  1.03E-09  8.80E-13  4.92E-09  6.20E-10  4.04E-16  8.44E-10  3.55E-33  1.66E-15  8.61E-20  1.65E-28  1.91E-10  2.60E-42  2.22E-17  4.08E-29  4.71E-09  2.08E-11  4.07E-16  3.07E-50  1.61E-20  3.73E-17  2.13E-18  5.20E-12  6.17E-21  8.30E-12  1.13E-11  7.38E-12  2.18E-68  1.12E-36  6.75E-18  2.32E-11  2.72E-09  7.46E-37  8.73E-16  7.17E-20  2.65E-29  2.56E-12  5.80E-104  1.42E-16  1.01E-08  2.10E-33  2.51E-15  1.12E-10  2.33E-17  2.63E-37  1.09E-10  4.98E-25  5.38E-16  1.11E-44  2.34E-09  9.57E-18  7.76E-35  5.75E-31  6.63E-09  1.27E-13  1.36E-54  1.04E-27  2.32E-11  1.33E-12  3.52E-22  2.32E-11  1.97E-09  2.32E-11  2.43E-31  7.03E-12  5.24E-24  1.65E-08  3.78E-13  2.21E-66  1.62E-39  7.65E-14  3.12E-37  3.41E-16  3.28E-30  1.16E-41  3.18E-08  2.60E-08  4.13E-17  9.33E-14  7.06E-09  1.40E-20  5.20E-11  2.62E-08  4.16E-15  5.15E-09  2.24E-22  1.43E-19  1.28E-12  1.45E-19  1.87E-12  1.99E-29  6.95E-11  2.02E-10  4.34E-10  1.79E-12  4.13E-19  6.20E-12  6.40E-09  3.30E-10  3.80E-08  8.30E-10  7.30E-09  4.20E-08  3.30E-09  7.70E-09  3.80E-10  1.40E-08  9.40E-09  2.20E-08  1.70E-08  3.90E-08  2.30E-10  8.60E-14  2.70E-08  5.00E-09  1.20E-12  6.60E-09  1.10E-14  2.40E-09  2.40E-08  9.40E-10  4.80E-10  6.70E-11  4.70E-08  4.30E-08  2.60E-08  3.40E-10  9.20E-09  2.40E-08  2.40E-13  4.80E-08  1.30E-18  5.40E-10  2.10E-08  6.70E-09  1.10E-09 | TRUE  TRUE  TRUE  TRUE  TRUE  TRUE  TRUE  TRUE  TRUE  TRUE  TRUE  TRUE  TRUE  TRUE  TRUE  TRUE  TRUE  TRUE  TRUE  TRUE  TRUE  TRUE  TRUE  TRUE  TRUE  TRUE  TRUE  TRUE  TRUE  TRUE  TRUE  TRUE  TRUE  TRUE  TRUE  TRUE  TRUE  TRUE  TRUE  TRUE  TRUE  TRUE  TRUE  TRUE  TRUE  TRUE  TRUE  TRUE  TRUE  TRUE  TRUE  TRUE  TRUE  TRUE  TRUE  TRUE  TRUE  TRUE  TRUE  TRUE  TRUE  TRUE  TRUE  TRUE  TRUE  TRUE  TRUE  TRUE  TRUE  TRUE  TRUE  TRUE  TRUE  TRUE  TRUE  TRUE  TRUE  TRUE  TRUE  TRUE  TRUE  TRUE  TRUE  TRUE  TRUE  TRUE  TRUE  TRUE  TRUE  TRUE  TRUE  TRUE  TRUE  TRUE  TRUE  TRUE  TRUE  TRUE  TRUE  TRUE  TRUE  TRUE  TRUE  TRUE  TRUE  TRUE  TRUE  TRUE  TRUE  TRUE  TRUE  TRUE  TRUE  TRUE  TRUE  TRUE  TRUE  TRUE  TRUE  TRUE  TRUE  TRUE  TRUE  TRUE  TRUE  TRUE  TRUE  TRUE  TRUE  TRUE  TRUE  TRUE  TRUE  TRUE  TRUE  TRUE  TRUE  TRUE  TRUE  TRUE  TRUE  TRUE  TRUE  TRUE  TRUE  TRUE  TRUE  TRUE  TRUE  TRUE  TRUE  TRUE  TRUE  TRUE  TRUE  TRUE  TRUE  TRUE  TRUE  TRUE  TRUE  TRUE  TRUE  TRUE  TRUE  TRUE  TRUE  TRUE  TRUE  TRUE  TRUE  TRUE  TRUE  TRUE  TRUE  TRUE  TRUE  TRUE  TRUE  TRUE  TRUE  TRUE  TRUE  TRUE  TRUE  TRUE  TRUE  TRUE  TRUE  TRUE  TRUE  TRUE  TRUE  TRUE  TRUE  TRUE  TRUE  TRUE  TRUE  TRUE  TRUE  TRUE  TRUE  TRUE  TRUE  TRUE  TRUE  TRUE  TRUE  TRUE  TRUE  TRUE  TRUE  TRUE  TRUE  TRUE  TRUE  TRUE  TRUE  TRUE  TRUE  TRUE  TRUE  TRUE  TRUE  TRUE  TRUE  TRUE  TRUE  TRUE  TRUE  TRUE  TRUE  TRUE  TRUE  TRUE  TRUE  TRUE  TRUE  TRUE  TRUE  TRUE  TRUE  TRUE  TRUE  TRUE  TRUE  TRUE  TRUE  TRUE  TRUE  TRUE  TRUE  TRUE  TRUE  TRUE  TRUE  TRUE  TRUE  TRUE  TRUE  TRUE  TRUE  TRUE  TRUE  TRUE  TRUE  TRUE  TRUE  TRUE  TRUE  TRUE  TRUE  TRUE  TRUE  TRUE  TRUE  TRUE  TRUE  TRUE  TRUE  TRUE  TRUE  TRUE  TRUE  TRUE  TRUE  TRUE  TRUE  TRUE  TRUE  TRUE  TRUE  TRUE  TRUE  TRUE  TRUE  TRUE  TRUE  TRUE  TRUE  TRUE  TRUE  TRUE  TRUE  TRUE  TRUE  TRUE  TRUE  TRUE  TRUE  TRUE  TRUE  TRUE  TRUE  TRUE  TRUE  TRUE  TRUE  TRUE  TRUE  TRUE  TRUE  TRUE  TRUE  TRUE  TRUE  TRUE  TRUE  TRUE  TRUE  TRUE  TRUE  TRUE  TRUE  TRUE  TRUE  TRUE  TRUE  TRUE  TRUE  TRUE  TRUE  TRUE  TRUE  TRUE  TRUE  TRUE  TRUE  TRUE  TRUE  TRUE  TRUE  TRUE  TRUE  TRUE  TRUE  TRUE  TRUE  TRUE  TRUE  TRUE  TRUE  TRUE  TRUE  TRUE  TRUE  TRUE  TRUE  TRUE  TRUE  TRUE  TRUE  TRUE  TRUE  TRUE  TRUE  TRUE  TRUE  TRUE  TRUE  TRUE  TRUE  TRUE  TRUE  TRUE  TRUE  TRUE  TRUE  TRUE  TRUE  TRUE  TRUE  TRUE  TRUE  TRUE  TRUE  TRUE  TRUE  TRUE  TRUE  TRUE  TRUE  TRUE  TRUE  TRUE  TRUE  TRUE  TRUE  TRUE  TRUE  TRUE  TRUE  TRUE  TRUE  TRUE  TRUE  TRUE  TRUE  TRUE  TRUE  TRUE  TRUE  TRUE  TRUE  TRUE  TRUE  TRUE  TRUE  TRUE  TRUE  TRUE  TRUE  TRUE  TRUE  TRUE  TRUE  TRUE  TRUE  TRUE  TRUE  TRUE  TRUE  TRUE  TRUE  TRUE  TRUE  TRUE  TRUE  TRUE  TRUE  TRUE  TRUE  TRUE  TRUE  TRUE  TRUE  TRUE  TRUE  TRUE  TRUE  TRUE  TRUE  TRUE  TRUE  TRUE  TRUE  TRUE  TRUE  TRUE  TRUE  TRUE  TRUE  TRUE  TRUE  TRUE  TRUE  TRUE  TRUE  TRUE  TRUE  TRUE  TRUE  TRUE  TRUE  TRUE  TRUE  TRUE  TRUE  TRUE  TRUE  TRUE  TRUE  TRUE  TRUE  TRUE  TRUE  TRUE  TRUE  TRUE  TRUE  TRUE  TRUE  TRUE  TRUE  TRUE  TRUE  TRUE  TRUE  TRUE  TRUE  TRUE  TRUE  TRUE  TRUE  TRUE  TRUE  TRUE  TRUE  TRUE  TRUE  TRUE  TRUE  TRUE  TRUE  TRUE  TRUE  TRUE  TRUE  TRUE  TRUE  TRUE  TRUE  TRUE  TRUE  TRUE  TRUE  TRUE  TRUE  TRUE  TRUE  TRUE  TRUE  TRUE  TRUE  TRUE  TRUE  TRUE  TRUE  TRUE  TRUE  TRUE  TRUE  TRUE  TRUE  TRUE  TRUE  TRUE  TRUE  TRUE  TRUE  TRUE  TRUE  TRUE  TRUE  TRUE  TRUE  TRUE | 0.000789383  0.001093564  0.001413553  0.000928716  0.001042217  0.000823636  0.001166355  0.000909493  0.000915181  0.001435715  0.000903723  0.00156274  0.000129598  0.0001116  0.000177461  0.00010842  0.000157752  0.000455662  9.12E-05  7.39E-05  5.66E-05  0.000275119  6.16E-05  0.000150691  9.48E-05  0.000172435  0.000116137  0.000169816  8.71E-05  0.000109243  6.71E-05  9.97E-05  6.27E-05  0.000297404  6.25E-05  7.54E-05  0.002530838  0.000233639  7.07E-05  7.69E-05  8.73E-05  6.83E-05  5.56E-05  0.000179314  8.67E-05  0.000156775  6.52E-05  0.000123676  7.05E-05  0.000111975  0.00023276  0.000151811  0.000169145  5.89E-05  8.80E-05  6.27E-05  0.000158687  5.99E-05  8.16E-05  0.000107545  6.89E-05  6.07E-05  6.16E-05  0.000763838  8.61E-05  0.000164256  0.000131407  0.000129352  0.000126348  0.000144831  9.14E-05  0.000118136  0.00018338  0.000289715  0.000109259  0.000682938  0.000207876  0.000205621  8.46E-05  0.000116379  0.000138746  0.000157164  0.000100281  6.88E-05  0.000162554  8.48E-05  6.85E-05  5.95E-05  5.59E-05  7.63E-05  7.09E-05  0.000128462  0.000151966  0.000104618  0.000265641  0.000279155  0.000513848  0.000107003  0.000118263  0.000107689  0.000311275  0.000190435  8.00E-05  0.000103451  9.47E-05  9.33E-05  5.39E-05  0.000283049  0.000103155  8.80E-05  5.83E-05  6.47E-05  6.44E-05  0.00011292  5.63E-05  9.53E-05  8.31E-05  9.03E-05  0.000293461  0.000200538  9.95E-05  0.000134864  6.98E-05  7.81E-05  9.14E-05  0.000213164  6.61E-05  0.00100538  9.85E-05  5.79E-05  8.73E-05  0.00035995  0.000205762  0.000265624  0.000112459  0.000134381  0.000101626  7.49E-05  8.65E-05  0.000302529  0.000170563  0.000143451  0.000134688  6.51E-05  7.95E-05  6.20E-05  8.00E-05  0.000162754  0.000142131  0.000239651  0.000126081  6.88E-05  8.18E-05  0.000213255  0.000124789  9.24E-05  7.46E-05  5.52E-05  7.97E-05  5.40E-05  0.00013395  0.000246158  6.97E-05  0.000255679  6.48E-05  0.000182144  0.000374309  0.000156911  0.000383441  7.74E-05  0.000124097  0.000447174  6.23E-05  0.000144421  6.75E-05  5.98E-05  9.56E-05  5.92E-05  0.000142853  0.000101933  0.000127736  0.000104702  9.66E-05  0.000116702  8.38E-05  0.00012309  6.79E-05  0.000123245  0.000156818  6.84E-05  5.45E-05  0.000260592  6.57E-05  9.24E-05  0.00021009  5.50E-05  0.000360295  9.61E-05  5.81E-05  7.62E-05  6.27E-05  0.00015354  0.000127835  7.84E-05  7.70E-05  6.31E-05  7.45E-05  0.00011699  0.000106577  6.56E-05  6.41E-05  5.70E-05  6.01E-05  5.74E-05  0.000218265  0.00123348  7.87E-05  0.000201581  7.16E-05  5.58E-05  6.36E-05  8.78E-05  9.20E-05  0.000505104  8.40E-05  8.08E-05  7.77E-05  5.95E-05  7.73E-05  0.000278875  0.000145851  0.0003401  0.000111123  0.000167297  6.36E-05  0.000280289  0.00011318  8.77E-05  7.01E-05  7.87E-05  7.52E-05  0.000268025  0.000498091  0.000160915  8.05E-05  6.10E-05  5.60E-05  5.56E-05  0.000111254  9.10E-05  8.39E-05  0.00026346  0.000259304  5.61E-05  0.000174883  6.34E-05  8.75E-05  7.65E-05  6.40E-05  8.63E-05  9.28E-05  0.000173387  8.15E-05  5.69E-05  6.86E-05  0.000170997  5.86E-05  0.000159645  6.49E-05  5.67E-05  0.000121458  0.000131134  0.000145465  7.54E-05  8.69E-05  0.000105081  5.85E-05  8.06E-05  8.81E-05  0.000416836  0.000277632  6.11E-05  7.21E-05  0.00034991  0.000152844  6.33E-05  7.07E-05  5.47E-05  7.61E-05  0.000120773  0.000311676  0.000125041  0.000498887  0.000111879  5.87E-05  0.000115191  0.000161259  0.000118661  0.000103268  8.68E-05  0.000111193  8.10E-05  6.20E-05  0.000159143  7.06E-05  0.000100961  6.35E-05  0.000237419  6.69E-05  6.81E-05  6.95E-05  9.10E-05  6.87E-05  0.000112171  0.000149929  8.03E-05  0.000110785  0.000147174  0.000277187  0.000124317  0.000200104  0.00012762  0.000146916  8.60E-05  8.79E-05  0.000160928  7.74E-05  0.000123092  0.000171251  0.000103925  7.62E-05  5.90E-05  0.000423364  5.32E-05  5.86E-05  0.000148614  6.19E-05  8.95E-05  0.000188157  6.09E-05  0.000153357  0.000157428  0.000101591  0.00013256  0.000106319  0.000155223  6.27E-05  8.71E-05  0.000178546  0.00015642  0.000114716  8.59E-05  6.45E-05  0.00046272  0.000154947  0.000111223  8.64E-05  0.000159641  0.000136388  9.16E-05  0.000129218  0.000408112  0.000193725  5.60E-05  7.27E-05  0.000112028  0.000121282  0.000110808  5.35E-05  0.000188991  6.31E-05  5.77E-05  5.41E-05  7.83E-05  9.61E-05  0.000147743  0.00023952  5.85E-05  7.28E-05  0.00019046  0.000117267  0.000464838  0.000142806  0.000354845  7.50E-05  0.000200731  8.00E-05  0.000109789  5.68E-05  0.000374871  9.09E-05  0.000128067  7.73E-05  0.000129246  0.0001121  5.90E-05  0.00011365  0.000342061  9.19E-05  0.000146574  0.000122954  0.000158302  0.000157692  0.000289666  0.000173689  8.38E-05  6.50E-05  0.000169355  8.22E-05  0.000186356  5.83E-05  6.56E-05  0.000241827  0.000123652  6.57E-05  5.78E-05  0.000156569  6.73E-05  0.000220327  6.34E-05  9.66E-05  9.64E-05  9.06E-05  5.59E-05  0.000132033  0.000109748  6.44E-05  7.08E-05  0.000102267  7.68E-05  0.000116149  0.000393352  6.22E-05  6.60E-05  9.11E-05  6.47E-05  6.97E-05  0.000116338  6.63E-05  0.000246238  0.000112781  0.000148389  0.000225698  7.09E-05  0.000333258  0.000124307  0.000224674  6.37E-05  8.40E-05  0.000114007  0.000411861  0.000152712  0.000127989  0.000142193  9.52E-05  0.000149981  8.37E-05  8.25E-05  8.51E-05  0.000536587  0.000310533  0.00013791  7.86E-05  6.27E-05  0.000335345  0.00011196  0.000153475  0.000216439  0.00011857  0.000886421  0.000123243  5.93E-05  0.000262645  0.000109405  7.20E-05  0.000128443  0.00029286  7.36E-05  0.000179329  0.000121201  0.000346626  6.40E-05  0.000126024  0.000274195  0.000441568  5.96E-05  9.70E-05  0.000419192  0.000203458  8.03E-05  9.06E-05  0.000163233  8.03E-05  6.36E-05  7.75E-05  0.000247188  8.80E-05  0.0001758  6.20E-05  9.01E-05  0.00053454  0.000322921  0.000104016  0.000286112  0.000115165  0.000222553  0.000318103  5.42E-05  5.77E-05  0.000131533  9.39E-05  5.71E-05  0.000148368  7.76E-05  5.64E-05  0.000113992  5.80E-05  0.000171745  0.000150124  8.81E-05  0.000172082  9.20E-05  0.000219692  0.00007722  7.71E-05  7.93E-05  8.95E-05  0.000147523  3.81E-05  2.70E-05  3.17E-05  2.45E-05  3.02E-05  2.65E-05  2.41E-05  2.79E-05  2.68E-05  3.15E-05  2.60E-05  2.64E-05  2.68E-05  2.57E-05  2.42E-05  3.33E-05  4.50E-05  2.49E-05  2.74E-05  4.06E-05  2.70E-05  4.89E-05  2.85E-05  2.50E-05  3.03E-05  3.10E-05  3.41E-05  2.40E-05  2.62E-05  2.51E-05  3.15E-05  2.67E-05  2.61E-05  4.30E-05  2.39E-05  6.22E-05  3.09E-05  2.55E-05  2.69E-05  2.97E-05 | 202.6531772  280.8293602  363.1193587  238.4565635  267.6294541  211.4540104  299.5440162  233.516355  234.9782586  368.8206325  232.0335053  401.5031543  58.35802286  50.25270553  79.91449599  48.82061477  71.0374438  205.2514562  41.0828498  33.25806719  25.46390042  123.9039056  27.73527486  67.85728756  42.66929315  77.65067427  52.29581588  76.47103767  39.22841193  49.1912371  30.19381219  44.90484803  28.21996533  133.9431033  28.15120381  33.96598146  1142.37807  105.2184482  31.81805138  34.63051143  39.30739076  30.75855556  25.03381615  80.7490147  39.02918379  70.59774193  29.34824033  55.69082495  31.74630174  50.42146046  104.822624  68.36213454  76.16887115  26.54205107  39.61339282  28.21937266  71.45851897  26.95212598  36.72150715  48.42655646  31.01934427  27.31892226  27.74643601  344.1743011  38.77907581  73.96693087  59.17266453  58.24701746  56.8943127  65.21816285  41.13573355  53.19601365  82.58049311  130.4792697  49.19839033  307.6967234  93.61369417  92.59799563  38.08901941  52.40451346  62.47762474  70.77278785  45.15509082  30.97789626  73.20035559  38.19860914  30.84475447  26.78934351  25.17967882  34.37534623  31.92136868  57.84631201  68.43172169  47.10817614  119.6344448  125.7222193  231.4742899  48.18208653  53.25327302  48.49113405  140.1925802  85.75788631  36.02089553  46.58276859  42.63308219  42.01024413  24.28811103  127.4763639  46.44940184  39.61779094  26.25755713  29.11677333  29.00592788  50.84694819  25.35407055  42.93038313  37.41764124  40.64370356  132.1670419  90.30856485  44.79211607  60.72955526  31.42209077  35.15063744  41.15579335  95.99571066  29.77285789  453.1186387  44.37359583  26.07390159  39.31223143  162.1227404  92.66154917  119.6267574  50.63913491  60.51213557  45.76101433  33.73696506  38.92689825  136.2519785  76.80741191  64.59681911  60.65032382  29.3260047  35.81181542  27.91126233  36.03208325  73.29060543  64.00208389  107.9267715  56.77408263  30.97470168  36.82260134  96.03682074  56.19209234  41.62313702  33.6043713  24.85199214  35.89819101  24.30167117  60.31801385  110.8575482  31.37259788  115.1466993  29.19220994  82.02380457  168.5923478  70.65867925  172.7068766  34.83726579  55.88031628  201.4262942  28.02940024  65.03363486  30.40968438  26.93973079  43.05992643  26.67700407  64.32751977  45.89913287  57.51926806  47.14626931  43.47948216  52.55003476  37.72462445  55.42678613  30.57726178  55.49673624  70.61713992  30.81884971  24.52122953  117.3597749  29.56619631  41.61441751  94.61119901  24.783666  162.2781076  43.29086842  26.1452416  34.31391842  28.21783156  69.14081845  57.56391792  35.31443021  34.68226535  28.41641282  33.53695471  52.67980577  47.99040116  29.5207077  28.84211845  25.65616841  27.04067643  25.83102152  98.29335095  556.0490329  35.44955361  90.77845012  32.24133359  25.1261164  28.61902944  39.54878613  41.40537475  227.5334496  37.82879254  36.36278254  35.00701139  26.81337632  34.79291042  125.5959881  65.67771993  153.1791311  50.03791052  75.33671543  28.6157034  126.2331992  50.96384184  39.48337749  31.54434042  35.43434021  33.87351497  120.7080001  224.3727305  72.46217411  36.24120444  27.47261845  25.19414938  25.02174986  50.09648315  40.97059056  37.76893815  118.6518997  116.7795585  25.25938811  78.75344449  28.56720492  39.402407  34.44560799  28.80490972  38.84544034  41.78866718  78.07935662  36.6912546  25.63843962  30.89350463  77.00281416  26.38932358  71.89022379  29.23361276  25.53582988  54.6918685  59.04964836  65.50369705  33.92878282  39.15032999  47.31685172  26.34044581  36.30598129  39.67074913  187.7547727  125.0359185  27.5278609  32.47861828  157.5990805  68.82738019  28.50326786  31.84030797  24.62996133  34.28509737  54.38341233  140.3730998  56.30545244  224.7315669  50.37812321  26.44172497  51.86950031  72.61732222  53.43243938  46.50051208  39.10167202  50.06905972  36.49092858  27.91374425  71.66415311  31.79502916  45.46145994  28.6135241  106.9211886  30.12443153  30.68166106  31.27936903  40.98370348  30.91301648  50.50972076  67.51411299  36.16504918  49.88559008  66.2735583  124.83547  55.97936551  90.11319367  57.46730451  66.15712885  38.7274628  39.5623567  72.46791766  34.85495197  55.42800461  77.11751392  46.79629622  34.29881834  26.58209463  190.6965386  23.95566233  26.36713439  66.92215013  27.87558329  40.29398634  84.73204244  27.41043734  69.05808226  70.89148043  45.74494234  59.69195173  47.87415896  69.89857719  28.23754211  39.23304088  80.40301267  70.43751332  51.65573204  38.6861139  29.04114358  208.4319911  69.77414449  50.08278109  38.92301327  71.88855706  61.41589568  41.23577916  58.18653589  183.8239705  87.23990439  25.23505488  32.72816644  50.44543968  54.61277415  49.89579351  24.069756  85.10765798  28.39878866  25.9710293  24.37257947  35.27059415  43.280963  66.52974758  107.8674352  26.32301512  32.759241  85.76936619  52.80481363  209.3863934  64.30610839  159.8224431  33.76145301  90.395498  36.03476938  49.43689802  25.5588509  168.8458059  40.93309719  57.66838357  34.80420696  58.19933325  50.47757651  26.58289679  51.17571904  154.0626101  41.35990358  66.00311576  55.36569443  71.28523311  71.01049736  130.4573636  78.21531495  37.7222489  29.26312117  76.26351251  36.99851027  83.92067901  26.24520704  29.51902357  108.9065943  55.68014162  29.58586881  26.02587715  70.50487475  30.29355465  99.22197915  28.56311179  43.47592087  43.39581412  40.78350981  25.17224866  59.4543492  49.41861553  29.01382336  31.89152862  46.04950555  34.59006332  52.30121305  177.1730516  27.98491192  29.71093592  41.01400861  29.15136159  31.38583842  52.38622634  29.85922518  110.8935296  50.78446336  66.82090495  101.6414962  31.92488305  150.0966198  55.97526736  101.1799613  28.69779357  37.80556893  51.33640831  185.513198  68.76771424  57.63334657  64.03000338  42.85712055  67.53794993  37.69452832  37.16729625  38.33070984  241.7232073  139.8579836  62.10119355  35.38758047  28.21259295  151.0368326  50.41442773  69.11143441  97.47085859  53.3913895  399.4571001  55.4957172  26.69602315  118.2848188  49.26391739  32.42987098  57.83772258  131.8963627  33.15913002  80.75584816  54.57636357  156.1191703  28.80825581  56.74820337  123.4877712  198.8999028  26.85764216  43.66813624  188.8164968  91.62395283  36.14171445  40.79525139  73.50625495  36.17570577  28.65686292  34.88026226  111.3215835  39.60612238  79.16619876  27.91971228  40.56408232  240.8006361  145.4391739  46.83700406  128.8563504  51.85798146  100.2247787  143.2687126  24.39290605  25.98501539  59.22949718  42.30300307  25.71361017  66.81146448  34.93510158  25.37619258  51.32983961  26.13295849  77.33981994  67.60205769  39.68102287  77.49149546  41.44574367  98.93613637  34.77029498  34.72652711  35.691396  40.31888222  66.43069904  17.50367339  12.43054156  14.59758508  11.25279036  13.89563456  12.2097568  11.0916302  12.8538567  12.31777301  14.46729928  11.97632202  12.14759089  12.31766378  11.80265871  11.14810643  15.32528036  20.68675269  11.43712134  12.60579522  18.66710735  12.43269701  22.4775557  13.11215192  11.48946985  13.93290882  14.26687784  15.68631516  11.02391788  12.0585284  11.56254971  14.48733353  12.29804155  12.00458769  19.7729262  10.98585805  28.62005668  14.20661654  11.74451771  12.38821376  13.66131438 |

SNP: single nucleotide polymorphism; IVs: instrumental variables; EA: effect allele; OA: other allele; eaf: effect allele frequency; se: standard error; explained variance given in percent;
