# Supplementary material for: Causal association between sarcopenia-related traits and osteoarthritis: A bidirectional 2-sample Mendelian randomization Study
Source: Medicine (Baltimore). 2025 Jul 11;104(28):e43069. doi: 10.1097/MD.0000000000043069 (PMC12263018; doi:10.1097/MD.0000000000043069)
Supplement: Supplementary file 3 [file medi-104-e43069-s003.docx]

| Exposure | SNP | EA | OA | eaf.exposure | | beta.exposure | | se.exposure | pval.exposure | mr_keep | R2 | F-statistics |
| --- | --- | --- | --- | --- | --- | --- | --- | --- | --- | --- | --- | --- |
| Hip OA | rs12040949 | T | C | | 0.3843 | | -0.0665 | 0.012 | 2.83E-08 | TRUE | 0.002092728 | 825.9935245 |
| Hip OA | rs11583641 | T | C | | 0.2764 | | -0.0811 | 0.0131 | 5.57E-10 | TRUE | 0.002630924 | 1038.978135 |
| Hip OA | rs4338381 | G | A | | 0.3681 | | -0.095 | 0.0121 | 4.37E-15 | TRUE | 0.004198473 | 1660.62892 |
| Hip OA | rs74767794 | G | A | | 0.3171 | | -0.0751 | 0.0126 | 2.56E-09 | TRUE | 0.002442661 | 964.4492109 |
| Hip OA | rs7571789 | C | T | | 0.5239 | | -0.0886 | 0.0117 | 3.26E-14 | TRUE | 0.003916012 | 1548.467399 |
| Hip OA | rs1835323 | T | C | | 0.3428 | | -0.0673 | 0.0123 | 4.56E-08 | TRUE | 0.002040791 | 805.4520804 |
| Hip OA | rs3774355 | A | G | | 0.3601 | | 0.0907 | 0.0121 | 8.20E-14 | TRUE | 0.003791227 | 1498.937225 |
| Hip OA | rs798748 | C | T | | 0.6183 | | 0.0715 | 0.012 | 2.50E-09 | TRUE | 0.002413034 | 952.7231656 |
| Hip OA | rs1913707 | G | A | | 0.3877 | | -0.0795 | 0.012 | 2.96E-11 | TRUE | 0.003000712 | 1185.450628 |
| Hip OA | rs12209223 | A | C | | 0.1032 | | 0.1558 | 0.0191 | 3.88E-16 | TRUE | 0.004493039 | 1777.664926 |
| Hip OA | rs2396502 | C | A | | 0.6018 | | 0.0842 | 0.012 | 2.12E-12 | TRUE | 0.003397877 | 1342.888069 |
| Hip OA | rs115740542 | C | T | | 0.0741 | | 0.1263 | 0.0224 | 1.60E-08 | TRUE | 0.002188865 | 864.021697 |
| Hip OA | rs80287694 | G | A | | 0.1131 | | 0.1093 | 0.0184 | 2.66E-09 | TRUE | 0.002396666 | 946.2452084 |
| Hip OA | rs60890741 | CA | C | | 0.1362 | | -0.1087 | 0.0185 | 4.50E-09 | TRUE | 0.002780221 | 1098.101572 |
| Hip OA | rs13300602 | G | A | | 0.4508 | | 0.0716 | 0.0119 | 1.65E-09 | TRUE | 0.002538461 | 1002.370607 |
| Hip OA | rs34687269 | T | A | | 0.4684 | | -0.0826 | 0.0117 | 1.67E-12 | TRUE | 0.003397754 | 1342.839455 |
| Hip OA | rs10492367 | T | G | | 0.19 | | 0.1518 | 0.0148 | 1.25E-24 | TRUE | 0.007092709 | 2813.568316 |
| Hip OA | rs79056043 | G | A | | 0.0503 | | 0.1625 | 0.0268 | 1.33E-09 | TRUE | 0.002522848 | 996.1900475 |
| Hip OA | rs11059094 | T | C | | 0.4777 | | 0.0759 | 0.0117 | 7.38E-11 | TRUE | 0.002874675 | 1135.515518 |
| Hip OA | rs12901372 | G | C | | 0.4669 | | -0.0783 | 0.0118 | 3.46E-11 | TRUE | 0.003052011 | 1205.778623 |
| Hip OA | rs62063281 | G | A | | 0.2229 | | 0.0964 | 0.014 | 5.30E-12 | TRUE | 0.003219371 | 1272.112316 |
| Hip OA | rs7222178 | A | T | | 0.1991 | | 0.0965 | 0.0146 | 3.77E-11 | TRUE | 0.002969848 | 1173.221165 |
| Hip OA | rs4252548 | T | C | | 0.022 | | 0.2785 | 0.0396 | 1.96E-12 | TRUE | 0.003337659 | 1319.00939 |
| Hip OA | rs2836618 | A | G | | 0.2613 | | 0.0876 | 0.0132 | 3.20E-11 | TRUE | 0.002962414 | 1170.275712 |
| Knee OA | rs17567417 | C | G | | 0.4698 | | -0.0655 | 0.0093 | 1.96E-12 | TRUE | 0.002137299 | 863.4377694 |
| Knee OA | rs12470967 | G | A | | 0.5746 | | -0.0584 | 0.0103 | 1.50E-08 | TRUE | 0.001667319 | 673.2556536 |
| Knee OA | rs1078301 | T | A | | 0.2685 | | 0.0679 | 0.0106 | 1.27E-10 | TRUE | 0.001811041 | 731.3948579 |
| Knee OA | rs56116847 | A | G | | 0.3563 | | 0.0612 | 0.0097 | 3.19E-10 | TRUE | 0.001718036 | 693.7698888 |
| Knee OA | rs4775006 | A | C | | 0.4114 | | 0.0578 | 0.0094 | 8.40E-10 | TRUE | 0.001617969 | 653.2959438 |
| Knee OA | rs35087650 | ATT | A | | 0.2589 | | 0.0694 | 0.0114 | 1.18E-09 | TRUE | 0.001848238 | 746.4448437 |
| Knee OA | rs8067763 | A | G | | 0.5936 | | -0.0566 | 0.0095 | 2.39E-09 | TRUE | 0.001545647 | 624.0490625 |
| Knee OA | rs143384 | G | A | | 0.4034 | | -0.0935 | 0.0095 | 4.77E-23 | TRUE | 0.004207967 | 1703.49245 |

**Supplementary Table 3. Detailed information of independent IVs for hip OA and knee OA**

SNP: single nucleotide polymorphism; IVs: instrumental variables; EA: effect allele; OA: other allele; eaf: effect allele frequency; se: standard error
